# The pro-export effect of subnational migration networks: new evidence from Spanish provinces

**DOI:** 10.1007/s10290-021-00423-4

**Published:** 2021-06-30

**Authors:** Anna D’Ambrosio, Sandro Montresor

**Affiliations:** 1 Polytechnic of Turin, Turin, Italy; 2 Gran Sasso Science Institute (GSSI), L’Aquila, Italy

**Keywords:** Gravity model, Migration, Subnational units, Poisson PML, Gamma PML, Fixed effects, F10, F14, F22, C52

## Abstract

The paper investigates the effect that subnational networks of immigrants and emigrants had on exports from Spanish provinces (NUTS3) over the period of 2007–2016 by integrating state-of-the-art advances in the gravity model literature. In particular, it allows for heterogeneity in provincial export capacity, which significantly reduces pro-export effects, and select the Poisson Pseudo-Maximum Likelihood as the most suitable estimator according to diagnostic tests. When both immigration and emigration are instrumented, the pro-export effect of immigrants found by previous studies vanishes and that of emigrants, instead, appears appreciable. The results obtained suggest that over the period that encompasses the double-deep crisis, immigrants did not show significant information and enforcement effects in the considered context, while the effects of emigrant demand for home-country goods may have been important. The prevalence of emigrant over immigrant effects appears attributable to a change in the composition of the migration stocks over the considered period of crisis.

## Introduction

Before the sudden halt brought about by the outbreak of the COVID-19 pandemic in the early 2020, migration flows towards OECD countries and across the world witnessed a persistent upsurge (OECD 2020b, https://www.migrationdataportal.org). The economic effects of migration have gained centrality in the academic debate, with major contributions addressing effects on employment, productivity, trade, and innovation.^1^ In particular, since the seminal works by Gould (1994) and Head and Ries (1998), a solid strand of literature has developed and has shown that migration contributes to trade in a significant way. Besides demanding home-country products, migrants attenuate the information and enforcement costs that, even in the ICT era, affect international trade (Rauch 2001; Anderson and van Wincoop 2004).^2^ Despite the rich empirical support for these effects, recent studies have started to raise concerns of a possibly overstated nexus (Parsons 2012; Burchardi et al. 2018). Moreover, results appear sensitive to the adopted empirical approaches—e.g., countries vs. subnational units of analysis, immigrants vs. emigrants, imports vs. exports, standard vs. differentiated commodities, similar vs. dissimilar countries)—uncovering the nuances of a still-open research issue. This is particularly so when we address the persistence of migration effects on trade during downturns in the business cycle, which has received little attention so far. Yet, addressing this issue appears very timely in light of the recent economic crisis caused by COVID-19 (Frankel and Romer 1999; Alcalá and Ciccone 2004; OECD 2020a; UNCTAD 2020).

Most studies analyzing the link between migration and trade (e.g., Gould 1994; Dunlevy and Hutchinson 1999; Rauch and Trinidade 2002) employ gravity models (Anderson and van Wincoop 2003; Chaney 2008; Head and Mayer 2014). Yet, the application of the gravity model to the analysis of the migration–trade link still reveals important gaps from both a theoretical and econometric perspective. We seek to fill these gaps. Using an integrated approach that draws on theoretical and methodological contributions from the frontier of the debate (Head and Mayer 2014; Correia et al. 2019, 2020; Weidner and Zylkin 2020), we investigate the case for an actual pro-export effect of migrants in a country characterized by large diasporas, like Spain, over a decade that has witnessed the interlinking of two economic crises (2007–2016). In doing so, we jointly consider three issues.

First, unlike the majority of previous studies, we simultaneously address the pro-export effects of both emigrants and immigrants. Indeed, when studying only one direction of migration the effects may be confounded and ascribed to the wrong underlying mechanism, especially in countries with large diasporas, like the one on which we focus.

Second, we refer to very disaggregated geographical units of analysis, i.e., Spanish NUTS3 regions (“*provincias*”). Due to the localized nature of knowledge spillovers (Audretsch and Feldman 1996; Rauch 2001) and the spatial heterogeneity in the distributions of both migration and trade (e.g., Peri and Requena-Silvente 2010; Bratti et al. 2014), the migration–trade link is arguably a localized phenomenon. Accordingly, the extant literature at the subnational level is quite developed.^3^ However, to the best of our knowledge, this is the first study to investigate the export effects of emigration (along with immigration) at the subnational level by allowing for subnationally heterogeneous export capacities. More precisely, and in line with recent contributions (Briant et al. 2014; Burchardi et al. 2018; Bratti et al. 2020), we allow for subnational heterogeneity in the “multilateral resistance” factors that inhibit the trade of provinces to any partner (i.e., in the so-called “multilateral resistance term” (MRT); Anderson and van Wincoop 2003).

Third, we contribute to the discussion on the delicate methodological choice of how to estimate migration-augmented gravity models of trade with panel data: an issue at the frontier of econometric “best practices” to consistently identify the determinants of international trade (Larch et al. 2019, p. 487). Indeed, the literature has long indicated an ideal candidate estimator for gravity models. This appears to be the Poisson Pseudo Maximum Likelihood (PPML) estimator with time-varying exporter and importer fixed effects and with time-invariant region-country fixed effects. Such an approach would address the inconsistency of heteroskedastic log-linear models estimated by OLS (Santos-Silva and Tenreyro 2006) and allow for heterogeneous MRTs while controlling for bilateral heterogeneity in unobservable trade barriers (Feenstra 2004; Baldwin and Taglioni 2007; Baier and Bergstrand 2007). Until recently, however, the literature had not provided a solution to the issue of separation in PPML models, which is particularly severe in the context of high-dimensional fixed effects, and for which maximum likelihood estimates may not exist or may be incorrect (Santos-Silva and Tenreyro 2010, 2011; Larch et al. 2019; Correia et al. 2019). Moreover, the asymptotic properties of PPML estimates with more than two-way fixed effects were still unclear (Weidner and Zylkin 2020). In this paper, we take advantage of recent econometric and computational advances (Correia et al. 2019, 2020; Weidner and Zylkin 2020) to improve the implementation of this approach. Moreover, taking stock of the simulation results by Head and Mayer (2014), who cast doubts on the use of PPML as a “workhorse” estimator for gravity models, we select the most suitable estimator through diagnostic tests that address the underlying distribution of the errors and the sources of potential misspecification (Head and Mayer 2014; Manning and Mullahy 2001; Santos-Silva and Tenreyro 2006).

We carry out our integrated analysis by focusing on exports. We study 5450 trading pairs, constituted by 50 Spanish provinces^4^ and 109 countries over the period of 2007–2016. We first work out the elasticity of exports to both immigration and emigration. Then, we revisit some stylized facts of the migration–trade link, like the role of institutional and language similarity between trade partners and the distinction between local and non-local effects of migration. Finally, we provide a first exploration of the extent to which the effects of migrants on exports has changed over time. Indeed, focusing on the 2007–2016 timeline, we provide a first endurance test of the trade effects of migration during the subprime mortgage crisis, the unfolding of the sovereign debt default crisis, and the subsequent recession.

Our results only partially confirm the available knowledge on the issue and offer some novel insights. When both directions of migration are retained and their endogeneity is addressed through an instrumental variable (IV) strategy, we do not find robust evidence of an immigration effect on exports. Exports from Spanish provinces appear mainly driven by emigrants, and the ability of immigrants to promote trade appears to deteriorate with the global financial downturn.

The remainder of this paper is organized as follows. In Sect. 2, we position our research in the extant literature. In Sect. 3, we present the data used for our empirical analysis, and in Sect. 3.1 we illustrate its methodological novelties. Section 4 presents our results, and Sect. 5 offers some concluding remarks. Appendix A details our data sources and variables, and Appendices B and C contain a set of robustness checks and heterogeneity analyses.

## Migrants and exports at the subnational level: the case for heterogeneous export capacities

The analysis of the trade impacts of migration has so far mainly concentrated on the effects of *immigrants* on trade in their recipient countries. Furthermore, when implemented at the subnational level, analyses have generally assumed that the trade capacities of the recipient units of investigation (i.e., regions) are homogeneous. Both choices entail problematic implications that we address by integrating the role of emigrants along with that of immigrants and by allowing for export capacities to be heterogeneous across subnational units of analysis.

### The pro-export effects of both emigrants and immigrants

The mechanisms through which migration can affect trade have been extensively studied over the last decades.^5^ Migrants typically move to a new location and preserve a relationship with their origin countries by creating and maintaining social and business networks that span across countries (Rauch 2001; Rauch and Casella 2001). Their embeddedness in these transnational networks is at the core of migrants’ capacities to reduce the so-called informal barriers to trade, that is, of the “network effects” they exert on trade. First, given their knowledge of customs, laws, markets, language, and business practices on the two sides of their migration route, migrants can help fill the information gaps between sellers and buyers, and in doing so they facilitate the realization of new business opportunities (*information effect*). Second, within their transnational networks, migrants can put in place implicit enforcing mechanisms (e.g., punishment, sanctions, and exclusions) for international contractual relationships and compensate for the weakness of institutional protection mechanisms (*enforcement effect*). A different kind of effect (*preference effect*) refers to migrants’ preferences for products from their homeland, which increases trade unidirectionally, with emigrants adding to the foreign demand for exports and immigrants increasing the domestic demand for imports (Hatzigeorgiou 2010; Parsons 2012).

While the literature has mainly focused on immigrants, the above mechanisms can be argued to apply to emigrants as well (see, e.g., Murat and Pistoresi 2009; Parsons 2012). In countries with large diasporas, there is no ex-ante reason to expect that information and enforcement effects are only due to inward rather than outward migration; and emigrant preference effects may be substantial drivers of exports that may confound the results if neglected. Hence, omitting the emigration side from the analysis may not only overstate the immigrant effects, but more importantly, it could also lead to wrongly attributing to information and enforcement what is in fact a preference effect.

In spite of these problematic implications, the emigration side of the migration–trade nexus has generally been neglected so far,^6^ mainly due to the lack of data, and especially in studies with a subnational focus. This is doubly unfortunate. From a subnational perspective, the effects of emigration could, in fact, operate differently from those of immigration. For example, Spanish emigrants may access knowledge exchanged within networks of co-nationals from provinces other than their own, with whom they share a language and social capital. Accordingly, they would promote the realization of trade opportunities not only with their province of origin but with Spain as a whole.

Another reason for their joint analysis is that emigrants and immigrants are likely complementary and may perform their bridging role in different contexts. Emigrant destination countries may substantially differ from immigrant countries of origin, for example, in terms of resource endowments, cultural habits, and institutional setups (Girma and Yu 2002; Dunlevy 2006), and they could follow distinct historical routes (Gould 1994; Rauch 2001). Accordingly, the trade contribution of emigrants could be higher or lower than that of immigrants, depending on the intensity of the existing barriers to trade (Rauch 2001). Furthermore, differences in tastes and human capital could translate into different effects on the trade of specific commodities and services (Rauch and Trinidade 2002; Peri and Requena-Silvente 2010; Briant et al. 2014).

In conclusion, in addressing the effect migrants can have on trade and, like in our empirical application, on exports, the joint analysis of both emigrants and immigrants is crucial to obtaining accurate results and drawing valid conclusions.

### The gravity model with subnationally heterogeneous export capacities

The information flows that account for a large portion of migrants’ pro-trade effects strongly rely on the business and social networks that migrants create. These are networks that operate mainly through direct interpersonal contacts and proximity (Rauch 2001). Given the tendency of new incoming immigrants to settle close to places where other immigrants have already settled (Altonji and Card 1991; Card 2001) and given the subnational heterogeneity in the economic structure of countries (Bratti et al. 2014), network effects can be expected to be heterogeneous across subnational units of analysis. In Spain, for example, at the beginning of our period of analysis (2007) seven provinces (Madrid, Barcelona, Alicante, Valencia, Malaga, Murcia, and the Balearic Islands) contained about 62% of the country-level immigrants, and eight provinces (Barcelona, Madrid, Valencia, Pontevedra, Zaragoza, Bizkaia, Gipuzkoa, A Coruña) accounted for 59% of exports. Emigrants were only slightly less concentrated, with about 60% originating from 9 provinces (Madrid, A Coruã, Pontevedra, Barcelona, Ourense, Asturias, Santa Cruz de Tenerife, Lugo, Valencia). These facts clearly indicate the polarizing role of the provinces of Madrid, Barcelona, and Valencia, but also the subnational heterogeneity in the distribution of immigrants, emigrants, and exports. On the basis of this evidence, subnational heterogeneity in the pro-trade effects of migrants is to be expected.

A subnational analysis of the pro-trade effects of migrants is indeed highly desirable. Investigating such a localized phenomenon as migration at the country level could, in fact, suffer from the Modifiable Areal Unit Problem (MAUP, Openshaw 1983), and the choice of the subnational level of analysis appears preferable. Furthermore, the reference to subnational observations increases data variability and mitigates concerns of spurious correlations affecting the relationship between trade and migration (Wagner et al. 2002; Bratti et al. 2014). For these reasons, the literature has progressively moved towards a finer geographic disaggregation in terms of units of analysis (for a review of studies at the national vs. subnational level, see Peri and Requena-Silvente 2010 and Felbermayr et al. 2015). Despite this wealth of studies, however, the trade implications of migrants, from a subnational perspective, have not yet been fully exploited.

As in the case of national units of analysis, analysis at the subnational level has developed through the advances in the gravity model of international trade by Anderson and van Wincoop (2003). Their “Multinational Resistance Term Revolution” (Head and Mayer 2014) led to an important extension of its standard “naive” formulation, mainly drawn on the analogy with Newtonian law in physics (Tinbergen 1962; Bergstrand 1985). Since then, country *i*’s exports to country *j*, $X_{ij}$, are not only assumed to be a positive function of their economic masses $Y_{i}$ and $Y_{j}$ and a negative function of their distance and of the relative transaction costs, $\phi _{ij}$;^7^ in addition, the “monadic” terms are adjusted by the average openness to trade of each trading partner, briefly, by their “Multilateral Resistance Terms” (MRTs). Denoting with $\Omega _i$ the average market size accessible to the exporting country and with $\Phi _j$ the average degree of competition of the importing one,^8^ the “structural” form of the gravity equation (Head and Mayer 2014) in a cross-sectional context is the following:^9^

1 $$\begin{aligned} X_{ij}=\frac{Y_i}{\Omega _i}\frac{Y_j}{\Phi _j}\phi _{ij} \end{aligned}$$

Following the previous equation, any change in bilateral trade barriers, encapsulated in the “dyadic” term $\phi _{ij}$, like their reduction entailed by migration, should be evaluated relative to the MRT, rather than in absolute terms (Anderson and van Wincoop 2003). Following Baier and Bergstrand (2007) and Baldwin and Taglioni (2007), the application of Eq. 1 to a panel context requires recognizing that most variables of interest, including the MRT, are time-varying.

With subnational units, and in the absence of subnationally disaggregated data on the destination of exports, as in our case, the gravity model becomes asymmetrical. In our case, the exporters are the NUTS3 Spanish provinces, while the importers are the destination countries. However, the interpretation of the terms in Eq. 1 remains remarkably similar. Indeed, as recently formalized by Bratti et al. (2020), the heterogeneous productivity of firms in different regions implies subnationally heterogeneous exporting capacities.^10^ In turn, subnationally heterogeneous productivity suits well the case of countries marked by a geographically fragmented production structure, such as Spain, and bears implications for the study of the migration–trade link. The average productivity of firms located in a given province is in fact not unrelated to bilateral migration stocks. Provinces with more productive firms may have a more dynamic structure of opportunities, attract more migrants from any origin country, and have lower emigration rates. The overall supply of immigrant labor, in turn, may affect productivity, wages, and the offshoring decisions of firms (e.g. Ottaviano and Peri 2006; Ottaviano et al. 2013) and can ultimately affect the accessibility-weighted exporting capacities of the exporter.

In light of the previous arguments, and in order not to commit the “gold medal mistake” of the gravity literature (Baldwin and Taglioni 2007), subnational and country-level studies alike need to account for the heterogeneity of the exporter-side MRT (Baier and Bergstrand 2007). In a subnational context, the MRT represents the province’s (weighted) capacity of export to any country in the world.^11^

In spite of this rich theoretical background, the estimate of subnational gravity models with heterogeneous exporter MRTs was only recently incorporated into panel data analyses (see Bratti et al. 2020). Briant et al. (2014) and Burchardi et al. (2018) include exporter effects, but in a cross-sectional framework. Bandyopadhyay et al. (2008) and Peri and Requena-Silvente (2010) use panel data but assume the term to be constant across regions in the same country, while Bratti et al. (2014) assume it to be invariant across the provinces (NUTS3) of the same more aggregated regional (NUTS2) level of analysis.

In an attempt to fill this gap, in the empirical application that we propose with panel data, we allow for subnationally heterogeneous export capacities at the NUTS3 level (*provincias*, referred to as “provinces”) rather than the NUTS2 level (*Comunidades Autonomas*, referred to as “regions”).

## Empirical application

**Fig. 1:**
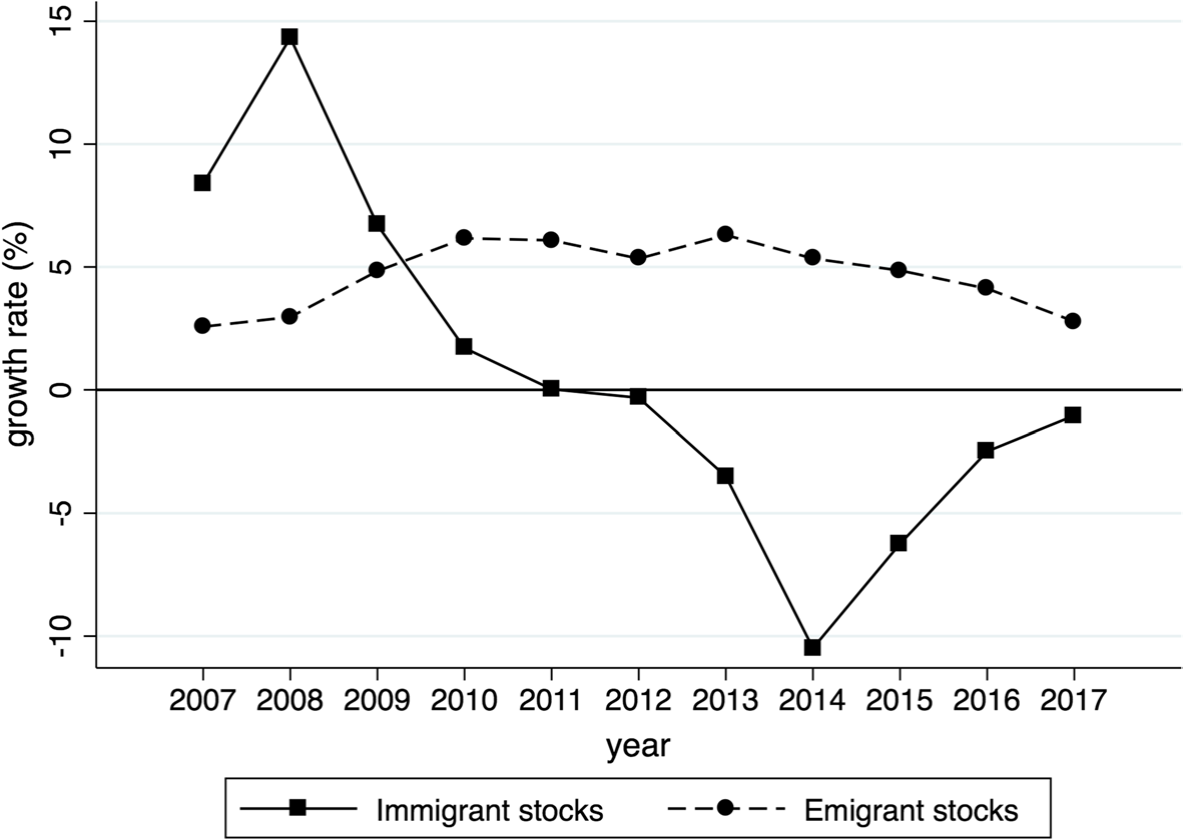
Growth rates of immigrant and emigrant stocks in Spain, 2007–2017. The total immigrant and emigrant stocks on which the growth rates are computed are obtained by aggregating our bilateral immigration and emigration stocks data by year. *Source* Own elaborations of Spanish National Statistical Institute (Instituto Nacional de Estadística, INE) data

We investigate the role of migration in driving the export performance of 50 Spanish provinces (NUTS3) towards 109 destination countries over the 10 years of 2007–2016. Compared to the previous study of the migration–trade link in Spanish provinces by Peri and Requena-Silvente (2010), we include a wider set of countries. We do this by drawing on the publicly available province-level dataset supplied by the Ministry of Economics and Competitiveness and by avoiding the elimination of dyads for which there are zero trade flows. Unlike Peri and Requena-Silvente (2010), who focused on the pre-crisis period (1998–2007), when immigration was booming, our analysis concentrates on a mainly negative phase of the business cycle, marked by the burst of the subprime mortgage crisis, the unfolding of the sovereign debt default crisis, and the subsequent recession, which heavily impacted the Spanish economy (e.g., Bentolila et al. 2012). Over this period, Spanish exports grew at an average rate of 4.1%, while emigration and immigration increased at an average rate of 4.7% and of 0.7%, respectively. The underlying patterns have, however, been very different, as illustrated in Fig. 1 for the countries in our sample. Export growth rates (not shown) faced a single substantial drop in 2009 and rapidly recovered. The growth of immigrant stocks slowed down over the entire period, taking negative values from 2011 onwards. Emigrant stocks increased at a stable pace over the considered period, but more strongly during the crisis years.

Given the particular trends that migration and exports revealed over this crisis period, studying their relationship represents an interesting exercise to evaluate the endurance of migration effects along the business cycle. To the best of our knowledge, this is the first study to perform such an analysis.

**Fig. 2:**
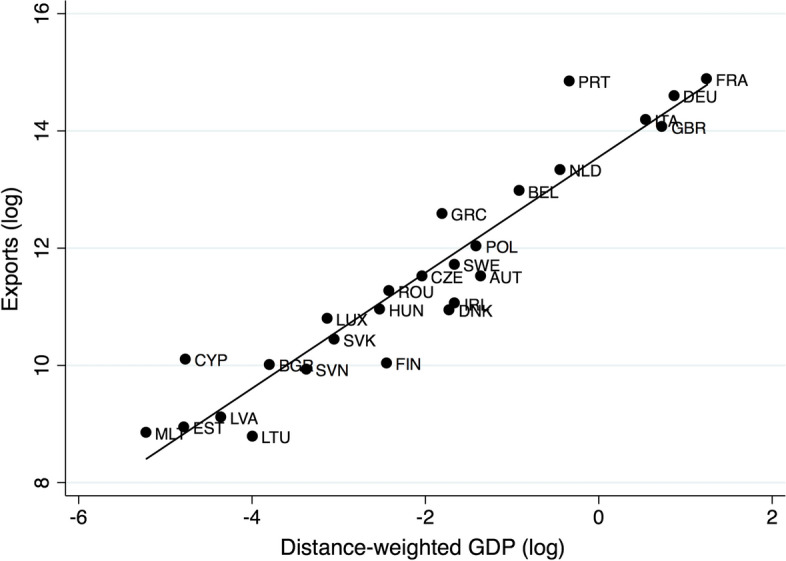
Gravity model and the exports of Madrid to EU countries, 2008. The figure plots the log exports of the province of Madrid to EU countries vs. the log of the ratio between each country’s GDP and its distance to Madrid. *Source* Own elaborations of MEIC and INE data

The dataset used for the empirical analysis is a balanced panel. Export data are retrieved from the official statistics of the Ministerio de Economia, Industria y Competitividad (MEIC) in Spain. For the sake of illustration, Fig. 2 represents the relationship between exports of the province of Madrid and the distance-weighted GDP of EU partner countries in 2008. The resulting picture is reassuring regarding the choice of the gravity model as an interpretative framework for the exports of Spanish provinces. The slope of the fitted line is 0.94, very close to one, in line with the stylized facts highlighted in the gravity literature and with the remarkable “law-like” behavior of international trade (Head and Mayer 2014). More details about our data sources, variables of interest, and summary statistics for these are provided in Appendix A.

### Econometric strategy

The specification that we employ to estimate the gravity model in Eq. 1 follows Baier and Bergstrand (2007) and Baldwin and Taglioni (2007) and includes a vector of country-year effects, $\theta _{\mathbf {jt}}$, and a vector of province-year effects, $\omega _{\mathbf {it}}$. Even by including these fixed effects, preferential ties linking specific dyads could still confound the estimation of the migrant effects. Historical reasons, including colonial history, past migration, and geography and transport infrastructure, may be responsible for tighter trade relationships between specific pairs, but also for larger bilateral migration stocks. In this case, the estimated effects of migration would also capture the role of history and geography (Briant et al. 2014; Burchardi et al. 2018). This limitation affects, for instance, the recent specification by Bratti et al. (2020). In order to address this issue, we thus include a further set of region-country fixed effects, $\eta _{\mathbf {rj}}$, which capture most of the time-invariant heterogeneity across dyads (Baier and Bergstrand 2007; Baldwin and Taglioni 2007). We cannot include dyadic (i.e., province-country) fixed effects, as the arguments by Baier and Bergstrand (2007) imply, due to the low residual variation in the data. Indeed, province-year, country-year, and province-country fixed effects explain between 90% and 98% of the variation in our dependent variable, depending on the estimator. In order to capture part of the residual pair-level heterogeneity, we further include bilateral distance, $Dist_{ij}$, between province–country dyads.^12^

On the basis of the previous choices, our identification strategy ultimately draws on two main sources of variation: the cross-sectional variation between provinces in the same region–country pair and the time variation within the region–country pair, which is not explained by country- and province-specific shocks. We are confident that this approach allows us to control for most confounding factors that would pose a threat to the internal validity: most importantly, that the more economically dynamic provinces within a given region are simultaneously the strongest exporters and the strongest attractors of migrants. At the same time, our identification strategy allows us to exploit the time and cross-sectional variation in the data, which is relevant for understanding the phenomenon.

We augment the resulting three-way fixed-effects gravity model by adding our variables of interest: the stock of immigrants from country *j* living in province *i* at time *t* $({\text {Immi}}_{ijt}$) and the stock of emigrants from province *i* living in country *j* at time *t* (${\text {Emi}}_{ijt}$). We log-transform both variables and add one unit to each of them to address the indeterminacy of the log of zeros. Furthermore, in order to control for possible non-linearities associated with this transformation, we add two dummy variables: “No Immigrant” ($NI_{ijt}$) and “No Emigrant” ($NE_{ijt}$). Each of the two dummies is equal to one if the immigrant (emigrant) stock from (to) country *j* to (from) province *i* in year *t* is equal to zero, and zero otherwise. Thus, our benchmark econometric model is the following:

2 $$\begin{aligned} X_{ijt} & = ({{\text {Y}}_{it-1}\times {\text {Y}}_{jt-1}})^{b_1}{\text {Dist}}^{b_2}_{ij}({\text {Immi}}_{ijt-1}+1)^{b_3}({\text {Emi}}_{ijt-1}+1)^{b_4}\nonumber \\&\times {\text {NI}}_{ijt-1}^{b_5}{\text {NE}}_{ijt-1}^{b_6}e^{(\omega _{\mathbf {it}}+\theta _{\mathbf {jt}}+\eta _{\mathbf {rj}}+\varepsilon _{ijt})}, \end{aligned}$$

where, besides the variables that we have already defined, $\varepsilon _{ijt}$ is a random error term with standard properties.

As the extant literature has highlighted, the choice of the most suitable estimator for Eq. 2 is not trivial. An intertwining set of econometric issues arise, which we address in the following subsections.

#### Zero trade flows and heteroskedasticity

A non-negligible share of the bilateral export values in our sample—about 7.5%—are zeros. The estimates of a standard OLS log-linear specification would only be based on positive trade values, posing a problem of selection bias. Previous studies have addressed the issue by opting for a Tobit model, with an arbitrary zero or an estimated threshold (Wagner et al. 2002; Herander and Saavedra 2005).

More recently, the Poisson Pseudo-Maximum Likelihood (PPML) estimator, which naturally accommodates zero trade flows, was recommended by Santos-Silva and Tenreyro (2006) as a “workhorse” for gravity models. The PPML estimator is consistent even with over- or under- dispersion (Wooldridge 2002) and when the share of zeros is substantial Santos-Silva and Tenreyro (2011).

PPML, and other estimators with the dependent variable in levels, is also recommended when the error term is heteroskedastic (Santos-Silva and Tenreyro 2006). In this case, log-linearizing the gravity equation to estimate it by OLS introduces a bias (see also Manning and Mullahy 2001; Blackburn 2007). A violation of the homoskedasticity assumption will, in general, lead to the expected value of the log-linearized error term in the log-linear transformation of the gravity model being dependent on the covariates. In other words, the conditional mean of the log of the errors will depend on both their mean and on the higher-order moments of their distribution. With heteroskedasticity, this will be correlated with the covariates, leading to inconsistent OLS estimates.

In partial contrast with these arguments, Head and Mayer (2014) show that relatively common misspecifications of the conditional mean—that is, taking as linear an effect that is actually non-linear—can lead to a severe bias in the PPML estimates due to the higher weight that this estimator places on larger observations. In this case, the more flexible distributional assumptions of the Gamma PML (GPML) are more suitable.

According to Head and Mayer (2014), the choice between a Poisson and a Gamma PML estimator should draw on an analysis of the underlying distribution of the errors. Following this claim, in our application we select a suitable estimator by applying the procedure that Head and Mayer suggest. To the best of our knowledge, this is the first application of this test to the analysis of the migration–trade link. As a first step, we estimate the gravity model by PPML, OLS (applied to the log-linear model), and GPML. In particular, for the OLS estimates, the dependent variable is the natural log of the strictly positive values of exports, ${\text {In}}(X_{ijt})$; for those by PPML, we employ the non-negative export values in levels $X_{ijt}$; and for those by GPML, we use their strictly positive values $e^{({\text {In}}(X_{ijt}))}$.

As a second step, we then perform a Park test for heteroskedasticity on the OLS estimates. With heteroskedasticity, we select the estimator via the “MaMu test”, discussed by Head and Mayer (2014), drawing on Santos-Silva and Tenreyro (2006) and Manning and Mullahy (2001). The test focuses on the relationship between the variance and the conditional mean of the residuals obtained by each estimator: ${\text {var}}[X_{ij}|\mathbf {z}_{ij}]= h{\text {E}}[X_{ij}|\mathbf {z}_{ij}]^\lambda$, where $\mathbf {z}_{ij}$ is the vector of covariates. The test estimates the empirical value of $\lambda$ by regressing the squared residuals on the fitted values of each model. The distribution of the errors is estimated by OLS when applied to OLS residuals, by PPML when applied to the PPML residuals, and by GPML when applied to the GPML residuals (Manning and Mullahy 2001; Santos-Silva and Tenreyro 2006).^13^

The outcome of the test gets read as in the following. Values of $\lambda$ close to 2 reflect a constant coefficient of variation, which is compatible with the Gamma distributional assumptions and with a log-normal distribution. The most efficient estimators, in this case, are the homoskedastic OLS on logs—which is the MLE if the homoskedasticity assumption is reasonable—and the Gamma PML.^14^ In this case, according to Weidner and Zylkin (2020), the Gamma PML with three-way fixed effects will also be consistent. If $\lambda$ is instead closer to 1, generalizing the Poisson distributional assumptions (Manning and Mullahy 2001), the Poisson PML is to be preferred as OLS will be inconsistent due to heteroskedasticity and Gamma PML will suffer from an incidental parameters problem.

As a third and final step, in order to corroborate the choice of the estimator based on the previous test, we run the Ramsey (1969) RESET tests on each estimation method, aiming to detect possible misspecifications in the conditional means. This could, for instance, arise from non-constancy in the covariates (Head and Mayer 2014).

#### Separation and incidental parameter problems

In our setting, the choice of the estimator that we discussed in the previous subsection is further complicated by the inclusion of three-way fixed effects. Maximum likelihood estimates in count data models—as well as more generally in non-linear models—may actually not exist if there is a problem of “separation” (Santos-Silva and Tenreyro 2010; Correia et al. 2019). In this case, the log likelihood increases monotonically as one or more coefficients tend to infinity. As a result of this, the log-likelihood cannot be maximized for any finite coefficient estimate and estimation algorithms fail to converge or yield incorrect estimates. This happens, for instance, when two regressors are collinear for the subsample of positive values of the dependent variable or, more generally, when the conditional mean is specified in such a way that its image does not include all the points in the support of the dependent variable. The problem is exacerbated by the inclusion of high-dimensional fixed effects. Indeed, the same problem has effectively hindered, until recently, the estimation of three-way gravity models by PPML (Larch et al. 2019). Studying the conditions governing the existence of a variety of generalized linear models, Correia et al. (2019) have recently shown how in the case of Poisson regression and even with high-dimensional fixed effects, the parameters of interest can usually be consistently estimated and problematic observations can be identified and dropped from the sample without affecting the validity of the estimates. Their method can be implemented in Stata via the ppmlhdfe routine (Correia et al. 2020).

A further estimation issue relates to the potential incidental parameters problem affecting three-way PPML and GPML estimates. This issue was recently addressed by (Weidner and Zylkin 2020). As for PPML, the absence of an incidental parameters problem in Poisson regressions with one-way fixed effects is a well-known result (Cameron and Trivedi 2015) that was shown to carry over to two-way models (Fernández-Val and Weidner 2016). However, while the three-way PPML estimator is also generally consistent, it is not unbiased as it suffers from an asymptotic incidental parameter bias of order 1/N. This bias affects the asymptotic confidence intervals in fixed-T panels, causing them to not be correctly centered at the true point estimates; the cluster-robust variance estimates are also downward biased. Weidner and Zylkin (2020) propose an algorithm to correct for this bias, ppml_fe_bias, which we implement here.

Weidner and Zylkin (2020) also study bias and consistency in Gamma PML models with high-dimensional fixed effects. The GPML with one-way fixed effects is free from the incidental parameters problem (Greene 2004), but this result carries over to the three-way GPML only if the conditional variance is correctly specified, that is, if it is proportional to the square of the conditional mean. Otherwise, the Gamma PML suffers from an incidental parameters problem, unlike the Poisson PML, that remains consistent under more general conditions. Weidner and Zylkin (2020) also provide a modified version of the algorithm in Correia et al. (2019) to estimate GPML models with high-dimensional fixed effects. This is contained in the Stata routine gpmlhdfe.

In what follows, we draw on these contributions to implement the selection of the estimator recommended by Head and Mayer (2014).

#### Endogeneity

Another issue that may affect our estimates is, of course, endogeneity. Even if we include large sets of fixed effects, our estimates may still suffer from the omission of bilateral time-varying variables or reverse causality. It has been argued that the direction of causality runs from migration to trade, as migration is generally driven by factors like family reunifications, wage differentials, and pre-existing co-ethnic communities (Gould 1994; Munshi 2003; Mayer 2004; Jayet and Ukrayinchuk 2007); yet, we cannot rule out the problem *a priori*.

We address this issue with an instrumental variables approach. To instrument immigration stocks, we resort to a slight modification of the standard shift-share instrument drawn from the labor economics and economic geography literature (see, e.g., Altonji and Card 1991; Card 2001; Ottaviano and Peri 2006). We move from the fact that new immigrants tend to co-locate where their co-nationals have previously settled, as the presence of co-ethnic networks decreases settlement costs and facilitates access to jobs and services. The remarkable path dependency in immigrant settlement ensures that immigrant stocks can typically be very accurately predicted by this instrument. On the other hand, pre-determined shares are arguably unrelated to current unobserved shocks affecting the outcomes of interest. This motivates the popularity of the “immigrant enclave” or “past settlement” instrument in a variety of settings (Jaeger et al. 2018), including in the migration–trade link literature. In this framework, exogenous shocks to the supply of immigrants from country *j* in year *t* (the “push” factor of immigration) affect the trade of provinces differently depending on the initial share of immigrants from country *j*.

We first build up weights for each country–province pair *ij* by using the share of immigrants from country *j* residing in province *i* over the total immigration from country *j* in a base year. These weights are then multiplied by the overall immigration stocks from country *j* to Spain in year *t* to obtain the imputed stocks. The main difference between our instrument and the standard shift-share approach is that by imputing bilateral stocks, we do not aggregate the imputed stocks by province. This is the procedure followed by other studies employing gravity models, such as Peri and Requena-Silvente (2010) and Bratti et al. (2014).

To minimize the risk that the initial shares are correlated to current unobservable shocks affecting trade flows, we construct the shares using the most remote year for which immigrant stocks data are available, i.e., 1991. As commonly argued in the literature (e.g., Hunt and Gauthier-Loiselle 2010; Autor et al. 2013), if these shocks are serially correlated, the exclusion restriction is more likely to hold with more remote shares. Moreover, the 1991 data are census-based and provide better coverage of immigration stocks than more recent intercensal estimates. The downside of using long-lagged shares is that a relatively large number of pairs turn out to have zero weight—whenever no immigrants from country *j* settled in province *i* in 1991. This is indeed the case for about 15% of the pairs in our sample.

The availability of data on residential variation (*Estadística de Variaciones Residenciales*) from 1988^15^ allows us to construct an additional, flow-based, instrument for immigration. In this case, we compute the shares based on the average inflows of foreign-born and foreign nationals by province and country over the 1988–1998 period, i.e., before the immigration boom of the early 2000s took place. The longer time coverage of this additional instrument should better address the issue of zero shares. With this additional instrument, we can flank our baseline just-identified 2SLS with an overidentified model and test for overidentification restrictions in a similar spirit to Briant et al. (2014).

Turning to emigration, we cannot construct a similar instrument due to the lack of historical data on Spanish emigration. Moreover, until 2006, the level of detail in residential variation data is also relatively poor for what concerns transfers to foreign countries. Hence, we propose an original procedure that reverses the logic of the recent works by Basile et al. (2018) and Beine and Coulombe (2018),^16^ and impute bilateral emigration based on aggregate residential inscription cancellations. In particular, we approximate the “push” factors driving emigrants to leave their province of origin with the overall cancellations in province *i* that involve a move to any foreign country. We proxy the “pull” factors leading emigrants to target a specific destination country with the overall cancellations that involve a move from any Spanish province to country *j*. In both cases, we assume that bilateral flows have negligible weight over aggregate outflows.

We denote with $w_{it}$ the share of residential cancellations from province *i* over the total cancellations at time *t* and with $w_{jt}$ the share of residential cancellations directed to country *j* over the total cancellations at time *t*. We use the product of $w_{it}$ and $w_{jt}$ to reweigh the total stocks of emigrants at time *t*, $Emi_t$. From this, we subtract bilateral stocks of emigrants $Emi_{ijt}$ to avoid perfect multicollinearity with the time-varying fixed effects and to mitigate concerns that the computation of the overall stocks was based on possibly endogenous bilateral stocks. The resulting instrument is ${\text {Emi}}^{\mathrm{imputed}}_{ijt}=w_{it}w_{jt}({\text {Emi}}_{t} - {\text {Emi}}_{ijt})$.

This way of accounting for “push” and “pull” factors is similar to Burchardi et al. (2018). However, data constraints prevent us from integrating any of the “recursive” factors that they use to account for persistent emigrant settlements from a specific province to a specific country over a long period of time. Nonetheless, drawing on the panel structure of our data, our 2SLS estimates include the three sets of province-year, country-year, and region-country effects (along with distance), as in all of our main specifications. Hence, our instrument will account for time-invariant ties between specified region–country dyads.

The popularity of the shift-share instruments has triggered many recent contributions to highlight their shortcomings.^17^ Most relevantly for our application, Jaeger et al. (2018) have noted that when the country-of-origin mix of immigrants is stable over time, the shifters are serially correlated. Hence, the instrument will correlate with its lags and the resulting estimates will conflate the short- and long-run effects of migration. Similar issues affect the other instrument that has been employed in the migration–trade link literature, i.e., the one based on the gravity model of migration employed by Bratti et al. (2020). These limitations also affect our proposed instrument for emigration. To disentangle the short-run from the longer-run adjustment to immigration, Bratti et al. (2020) propose instrumenting current and past migration jointly with the shift-share instrument and its lag. To yield valid estimates the two instruments must differ, hence there must be some innovation over time in the countries of origin.

This is indeed a limitation of our application. In our sample, the composition of immigrants does not provide sufficient variation to allow us to distinguish the consequences of current and past immigration, similarly to what the authors find for recent decades in the US. Despite the drop in immigration rates associated with the crisis, they remain highly serially correlated (correlation coefficients for all lag lengths $>0.88$). Indeed, the drop in the overall immigration stocks is mainly due to a decrease in immigrants from Ecuador, Colombia, and Argentina, who nonetheless remain—by far—the most represented among immigrants over the entire period. This implies that our estimates will conflate the short- and long-run adjustment of trade to immigration: that is, immigrant information and enforcement effects, as well as the trade effects of the longer-run adjustment of the local system to migration. As suggested by Bratti et al. (2020), migration may indeed affect export competitiveness via wage and productivity effects.

## Results

### The pro-export effect of migration

Based on our econometric strategy, Table 1 reports the results of the three estimators of the gravity model, including both immigrants and emigrants and allowing for heterogeneous MRTs at the province level.^18^ Starting with the building blocks of the gravity model, the product of per-capita GDP is collinear with the province-year and country-year fixed effects and is thus omitted.^19^ As for the distance variable, it has the expected negative effects in the OLS and GPML estimates, while its effect is insignificant, conditional on the bilateral region–country effects, according to the Poisson estimates. As previously mentioned, because the region-country effects absorb most of the pair-level variation, including distance or excluding it leaves the results virtually unaffected (see Appendix Table 9).

Coming to our focal migration variables, their point estimates are similar across the different estimators and specifications.

When included separately, both immigrants and emigrants positively and significantly affect provincial exports. On the other hand, the results change when they are included jointly, as expected. With the sole exception of the OLS estimates, a significant and positive effect on provincial Spanish exports is revealed only by emigrants, while the effect of immigrants turns out to be non-significant. Importantly, the positive yet comparatively small correlation between immigration and emigration stocks implies that the coefficient of each of these variables is somewhat overestimated when they are included separately. The non-significant or only mildly significant effects of ${\text {NI}}_{ijt-1}$ and ${\text {NE}}_{ijt-1}$ also reassure us that having added one unit to the migration variables does not alter the results. Across the considered estimators, a 10% increase in the emigration stock towards a certain country is found to increase exports by about 0.9% on average.

This is a first interesting result of our application. The result is original with respect to previous studies at the country level, which have found that the “impact of emigrant networks on exports coexists with a positive and significant impact of immigration on exports” (Hiller 2014, : 698).

As we stressed in Sect. 2.2, allowing for heterogeneous export capacity at the province level is a crucial choice to obtain accurate estimates in the gravity model at stake. In support of this argument, in Appendix B.2 we study whether the assumption of heterogeneous export capacity at the province level yields statistically different implications from less-demanding approaches. Results clearly indicate that ignoring this heterogeneity dramatically overstates both the immigrant and emigrant effects, supporting our theory-based approach.

**Table 1:** Pro-export effect of immigrants and emigrants: subregionally heterogenous MRTs

	(1)	(2)	(3)	(4)	(5)	(6)	(7)	(8)	(9)
	PPML			OLS			GPML		
$${\mathrm{ln}}({\text {Immi}}_{ijt-1}+1)$$	0.077$$**$$		0.053	0.064$$***$$		0.052$$**$$	0.036$$*$$		0.023
	(0.039)		(0.037)	(0.022)		(0.022)	(0.018)		(0.019)
$${\mathrm{ln}}({\text {Emi}}_{ijt-1}+1)$$		0.115$$***$$	0.096$$**$$		0.103$$***$$	0.091$$***$$		0.101$$***$$	0.097$$***$$
		(0.041)	(0.038)		(0.026)	(0.027)		(0.023)	(0.023)
${\text {NI}}_{ijt-1}$	− 0.104		− 0.133	0.046		0.022	− 0.022		− 0.047
	(0.092)		(0.091)	(0.068)		(0.068)	(0.054)		(0.054)
${\text {NE}}_{ijt-1}$		0.005	$-$0.000		0.043	0.037		0.064$*$	0.063$*$
		(0.067)	(0.068)		(0.043)	(0.043)		(0.036)	(0.036)
$${\mathrm{ln}}({\text {Dist}}_{ij})$$	− 0.117	0.009	0.022	−1.427$$***$$	−1.323$$***$$	−1.386$$***$$	−1.020$$***$$	− 0.927$$***$$	− 0.952$$***$$
	(0.350)	(0.362)	(0.363)	(0.364)	(0.363)	(0.361)	(0.319)	(0.319)	(0.315)
N	54,250	54,250	54,250	50,210	50,210	50,210	50,210	50,210	50,210
Province-year effects	Yes	Yes	Yes	Yes	Yes	Yes	Yes	Yes	Yes
Country-year effects	Yes	Yes	Yes	Yes	Yes	Yes	Yes	Yes	Yes
Region-country effects	Yes	Yes	Yes	Yes	Yes	Yes	Yes	Yes	Yes

Standard errors clustered at the province-country level in parentheses

$$*$$$$p<0.1$$, $$**$$$$p<0.05$$, $$***$$$$p<0.01$$

### Picking the right estimator

According to Head and Mayer (2014), a scenario like the one presented in Table 1, where the three estimators yield largely similar results, is reassuring in terms of there being no signals of major misspecification. In particular, including or excluding zero trade flows leaves our results virtually unaffected.

Still, as we have mentioned above, the log-linear OLS estimates are inconsistent when there is heteroskedasticity. A standard Park regression of the squared OLS residuals on the covariates (Table 15) confirms this suspicion: the variance of the residuals actually increases with the increase in both the immigrant and emigrant stocks and it decreases with the distance variable; furthermore, the variance is also, on average, smaller for provinces with no immigrants. Hence, with our data, Poisson and Gamma PML estimators should be preferred over OLS. This is confirmed by the “MaMu test” reported in Table 2.

The estimated value of $\lambda$ in Equation 13 is about 1.7 for OLS, about 1.3 for PPML, and 1.9 for GPML. In the latter case, the confidence intervals for the $\hat{\lambda }$ include 2. This implies that, provided that the conditional mean is well specified, the high-dimensional fixed-effects Gamma model that we implemented does not suffer from an incidental parameters problem, similarly to the fixed-effects Poisson model Weidner and Zylkin (2020). Moreover, while the $\hat{\lambda }$ for OLS and PPML are neither precisely 2 nor precisely 1, they seem to satisfy the distributional assumptions of their underlying estimators reasonably well. According to these estimates, the GPML would seem to be the most efficient estimator for our data. However, because the $\hat{\lambda }$ estimated for the OLS and PML residuals are both significantly below 2, the PPML estimator should be preferred over the OLS (see Head and Mayer 2014). In brief, the results of the MaMu test support the implementation of either the PPML or the GPML.

**Table 2:** MaMu and RESET tests

Model	PPML residuals	OLS residuals	GPML residuals
*Manning and Mullahy (MaMu) test on the underlying distribution of the errors*			
$${{\text {In}}(\hat{\mu })}$$	1.298$$***$$	1.697$$***$$	1.904$$***$$
	(0.047)	(0.005)	(0.059)
*RESET tests*			
Squared linear prediction	− 0.007	− 0.020	− 0.216
*p*-value	0.2418	0.001	0.000
N	54,250	50,210	50,210

Heteroskedasticity-robust standard errors in parentheses. $$*$$$$p<0.1$$, $$**$$$$p<0.05$$, $$***$$$$p<0.01$$

In the bottom panel of Table 2, we report the coefficients and* p*-values associated with a set of Ramsey (1969) RESET tests on each estimation method, again following Santos-Silva and Tenreyro (2006). The null hypothesis of the correct specification of the conditional mean cannot be rejected in the case of the Poisson PML, while it is rejected in the case of the OLS estimates and the Gamma PML.^20^

In conclusion, on the basis of the previous diagnostic test, the Poisson PML estimator emerges as the most suitable one for addressing our focal issue. On this basis, we infer that conditional on emigrant stocks, immigrants into Spanish provinces do not significantly increase the exports of these provinces towards the immigrants’ home countries. On the other hand, we detect a positive and significant effect of emigrants on Spanish provinces’ exports. Conditional on immigration stocks, a 10% increase in the emigration stocks of Spanish provinces increases exports to their countries of expatriation by almost 1% (Table 1). The emigrant effects on exports incorporate both a network effect (information and enforcement) and a preference effect, and their magnitude is comparable to previous estimates of the immigrant effect on imports. These previous estimates are generally larger than those for exports and lead to a corresponding increase of about 1.5% (see the meta-analysis by Genc et al. 2012). With respect to previous studies, our estimates are thus comparable but relatively smaller. Let us remember that this relatively conservative result is found by allowing for differential exporting capacities of provinces. Erroneously ruling out this heterogeneity would lead to much larger estimates of both the immigrant and emigrant effects (Table 10).

The result of an exclusive pro-export effect of emigrants is original and amenable to different interpretations. First of all, along a negative phase of the business cycle like the one we are considering, the opposing dynamics of immigration and emigration could have affected the composition of the respective stocks as well as the relative importance of the network and preference effects. While more refined data would be needed to ascertain the validity of this hypothesis with more accuracy, we argue that in the same period, the demand for home-country products (preference effect) expressed by emigrants could reasonably be the greatest, if not the only, relevant channel through which migrants can affect trade. Moreover, due to the wider set of mechanisms underlying their pro-export impact, the effect of emigrants may be easier to detect statistically. On more structural grounds, the same results can also be related to the level of productivity of the firms based in Spanish provinces. Previous evidence reveals that emigrants are more effective in promoting the trade of low-productivity firms, which have a lower capacity to enter into foreign markets (following Melitz’s selection argument) but a greater chance of overcoming trade barriers, with the help of emigrants, once they have entered (Hiller 2014).

Before taking these results as conclusive, three further steps are required to ensure that our estimates are reliable: i) ensuring that the estimates are robust to the bias-corrected method proposed by Weidner and Zylkin (2020); ii) addressing possible remaining sources of endogeneity; iii) and studying whether there is significant heterogeneity in the migrant effects, which could challenge the underlying assumption of constant elasticity in the Poisson PML model (Head and Mayer 2014). We will address these issues in turn in each of the following subsections.

### Bias-corrected PPML estimates

Weidner and Zylkin (2020) showed that the PPML with three-way fixed effects suffers from a peculiar type of incidental parameters problem: an asymptotic bias affecting confidence intervals and cluster-robust variance estimates. Based on their discussion, the size of the bias may be expected to be relatively small. Still, it is important to empirically appreciate whether it would lead to differences in our results. For this reason, we report the results of the bias-corrected three-way fixed effects PPML estimates in Table 3. The bias-corrected estimates are fully in line with the main results. This is so even with a bias of about 0.006 affecting the estimates of both our variables of interest ${\text {In}}({\text {Immi}}_{ijt-1}+1)$ and ${\text {In}}({\text {Emi}}_{ijt-1}+1)$, inflating the estimated coefficients by 10% and 6% and deflating the estimated standard errors by 8% and 9%, respectively. The insights derived from this approach still indicate a positive and significant effect of emigrants but not of immigrants on exports.^21^ Given the robustness of our results to the bias correction by Weidner and Zylkin (2020), we proceed with the standard uncorrected estimates in what follows.

**Table 3:** Bias-corrected PPML estimates

	Original	Bias	Adjusted SEs	Bias-corrected
${\text {In}}({\text {Immi}}_{ijt-1}+1)$	0.053 (0.050)	0.006	0.054	0.048 (0.054)
${\text {In}}({\text {Emi}}_{ijt-1}+1)$	0.095 (0.033)	0.006	0.036	0.089 (0.036)**
${\text {NI}}_{ijt-1}$	$-$0.133 (0.089)	$-$0.007	0.093	$-$0.127 (0.093)
${\text {NE}}_{ijt-1}$	$-$0.001 (0.064)	0.018	0.075	$-$0.019 (0.0754)

Observations: 54,250. Estimates include province-year, country-year, and region-country fixed effects. Standard errors are clustered by pair, using local de-biasing adjustment to account for estimation noise in the province-year and country-year fixed effects (Weidner and Zylkin 2020). $${*}p<0.10$$; $${**}p<0.05$$; $${***}p<0.01$$

### Instrumenting migration stocks

Table 4 reports our 2SLS estimates drawing on the instrumental variables discussed in Sect. 3.1.3.^22^

Columns (1) and (2) report the first-stage regressions. The F-statistics of both first-stage regressions, comfortably above the conventional value of 5, allow us to dismiss concerns about the potential weakness of our baseline instruments. As previously mentioned, this is a common result for instrumental variables such as our Altonij–Card-like instrument for immigration (column 1). Importantly, the F-statistics reported in column 2 are also reassuring in terms of the strength of our less-standard instrument for emigration.

Column (3) reports the second-stage estimates of the just-identified model, where the orthogonality of the instruments must be assumed. Results confirm the results of the Poisson PML and highlight an even larger role for emigrants than the one we identified in the baseline estimates, similarly to Bratti et al. (2014, 2020).^23^ The increase in the coefficient of emigration could be attributed to a measurement error: as discussed in Appendix A, ${\text {Emi}}_{ijt}$ likely underestimates the actual stocks as not all Spanish expats appear in the electoral registries of their host countries. By contrast, the data on residential cancellations may more rapidly capture the movements of Spanish nationals abroad.

In column (4), we report the results of the overidentified model that includes the additional instrument based on the 1988–1998 flows. Again, the F-tests on the first stage strongly reject the null hypotheses of weak instruments. The estimated 2SLS coefficients of immigration and emigration are remarkably similar to the ones in column (4). As for the overidentification test, the Hansen J statistic $\chi ^2(1)$ is 0.069 (*p*-value = 0.7927) and prevents us from rejecting the null hypothesis of instrument orthogonality.^24^ These results support the validity of our instruments and suggest that the positive effect of emigrants on trade detected in the previous section is robust to endogeneity concerns.

**Table 4:** 2SLS estimates

Model	Just-identified			Overidentified		
	First stage		Second stage	First stage		Second stage
Dep. var.	${\text {In}}({\text {Immi}}_{ijt-1}+1)$	${\text {In}}({\text {Emi}}_{ijt-1}+1)$	${\text {In}}({\text {X}}_{ijt})$	${\text {In}}({\text {Immi}}_{ijt-1}+1)$	${\text {In}}({\text {Emi}}_{ijt-1}+1)$	${\text {In}}({\text {X}}_{ijt})$
	(1)	(2)	(3)	(4)	(5)	(6)
$${\text {In}}({\text {Immi}}^{\mathrm{imputed}}_{ijt-1}+1)$$	0.136$$***$$	0.053$$***$$		0.114$$***$$	0.045$$***$$	
	(0.011)	(0.007)		(0.010)	(0.007)	
$${\text {In}}({\text {Emi}}^{\mathrm{imputed}}_{ijt-1}+1)$$	0.029	0.705$$***$$		$$-$$0.027	0.689$$***$$	
	(0.041)	(0.023)		(0.040)	(0.023)	
$${\text {In}}({\text {Immi}}^{\mathrm{imputed}^{88-98}}_{ijt-1}+1)$$				0.109$$***$$	0.036$$***$$	
				(0.009)	(0.006)	
${\text {In}}({\text {Immi}}_{ijt-1}+1)$			0.043			0.067
			(0.100)			( 0.077)
$${\text {In}}({\text {Emi}}_{ijt-1}+1)$$			0.325$$***$$			0.328$$***$$
			(0.085)			(0.085)
$${\text {In}}({\text {Dist}}_{ij})$$	1.215$$***$$	$$-$$0.264	$$-$$1.275$$***$$	1.151$$***$$	$$-$$0.281	$$-$$1.299$$***$$
	(0.373)	(0.262)	(0.388)	(0.347)	(0.257)	(0.377)
N	49,389	49,389	49,389	48,969	48,969	48,969
F-statistic	78.55	595.65		104.24	403.72	
Province-year effects	Yes	Yes	Yes	Yes	Yes	Yes
Country-year effects	Yes	Yes	Yes	Yes	Yes	Yes
Region-country effects	Yes	Yes	Yes	Yes	Yes	Yes

Standard errors clustered at the province-country level in parentheses

$$*$$$$p<0.1$$, $$**$$$$p<0.05$$, $$***$$$$p<0.01$$

### Sources of heterogeneity in the pro-export effect of migration

As the extant literature has largely shown (e.g., Girma and Yu 2002; Wagner et al. 2002), the trade effect of migrants usually interacts with standard trade determinants: first and above all, institutional and language commonality. Because PPML estimates place more weight on larger trade flows, it is important to study how sensitive our results are to heterogeneity along different dimensions. In order to check this, we carry out an additional set of analyses and distinguish trade partners by institutional and language commonality with Spain.^25^

The relative results, reported in Appendix C because of scope constraints, show that consistent with previous literature, the pro-export effect of emigrants is unambiguously driven by emigrants towards extra-EU countries. This actually confirms previous findings supporting the argument that differences in institutional settings increase transaction costs and make the role of migrants more salient in reducing them. On the other hand, we find stronger pro-trade effects of emigrants targeting Spanish- or English-speaking countries. This suggests that in the case of Spain, language commonality rather than similarity promotes trade. Quite interestingly, in our application institutions and language do not represent two sides of the same coin in transnational networking: unlike common institutions, language commonality adds to an emigrant’s ability to promote export. Drawing on the random encounter model proposed by Wagner et al. (2002),^26^ we could think that while trade barriers could be *per se* lower between countries speaking the same language, language commonality further eases migrants’ access to information about home- or host-country opportunities. In short, language commonality increases the probability that an emigrant has the capacity to facilitate a transaction.

We also study the role of geography and subnational concentration on migrants’ pro-trade effects (Herander and Saavedra 2005; Peri and Requena-Silvente 2010). In order to study the effects of geographic proximity, similarly to Herander and Saavedra (2005) we contrast the effects of immigrant and emigrant networks pertaining to a specific province with those of country-wide networks. Results in Appendix C show that a weakly significant pro-export effect of immigrants emerges for province-level networks only and that emigrant effects appear instead to be driven by both a localized and a country-wide component. This suggests that the exports of a given province *i* to a country *j* rely not only on the pro-trade effects of emigrants from those provinces but also on emigrants from provinces other than *i* who live in *j*. The networks through which immigrants and emigrants exert their pro-export effect in the case of Spanish provinces appears to be on a different scale: local and non-local, respectively. This represents, to the best of our knowledge, an additional novel result of this study.

As an additional implication of recognizing the role of geography in affecting migrants’ pro-export effects, we allow for these effects to be heterogeneous across provinces with different levels of migration concentration, as suggested by Herander and Saavedra (2005) and Peri and Requena-Silvente (2010).^27^ Consistent with Herander and Saavedra (2005), our results in Appendix C.3 indicate the existence of positive co-ethnic and inter-ethnic spillover effects from immigrants residing in high-concentration provinces, while those in low-concentration provinces do not reveal spillovers of this kind. On the other hand, no significant spillover effects emerge for emigrants, irrespective of the level of concentration. The results obtained by disaggregating provinces with high, medium, and low migration rates (Peri and Requena-Silvente 2010) are compatible with the arguments about the need for a minimum scale of immigration and emigration stocks for pro-trade effects to emerge. The positive effects of concentration are also confirmed when provinces are classified according to the values of a standard location quotient for immigration and emigration.

Finally, in the same Appendix where we develop the previous analyses of this subsection, we propose a methodology that, using an approximation with the data at our disposal, suggests that immigrant and emigrant effects on exports are stronger for provinces that are less specialized in the production of homogeneous intermediate goods. This is consistent with the original arguments by Rauch and Trinidade (2002). Migrant information effects would be more salient in the intermediation of trade transactions that concern differentiated goods and less salient for homogeneous goods, for which prices convey most of the relevant information.

All in all, while some heterogeneity in the effects emerges, the results of our estimators are similar to each other and do not raise substantial concerns that the greater weight given to larger observations by the PPML is biasing the results.

### Migrant effects over time

**Fig. 3:**
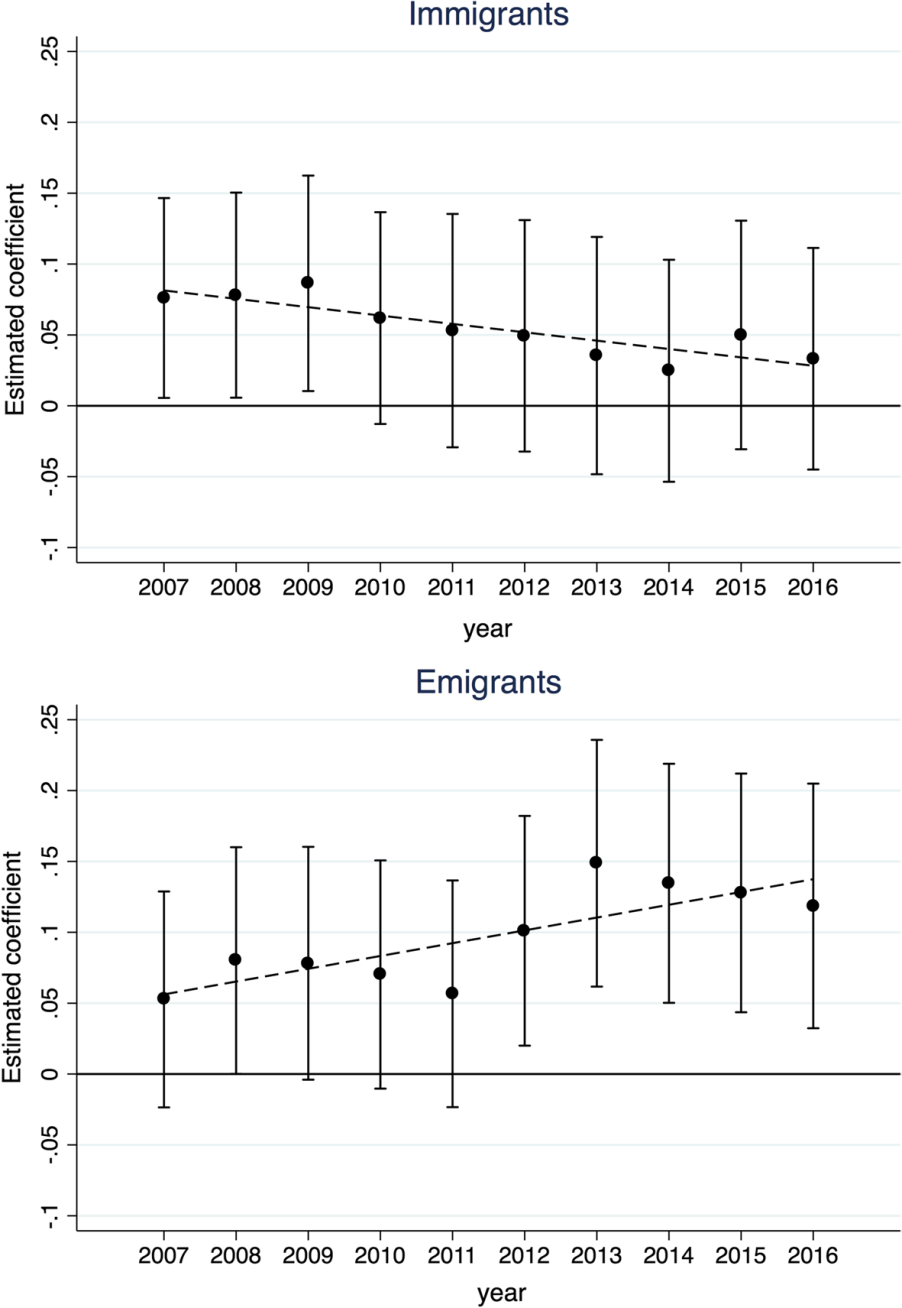
Time patterns of the migration effects

The dataset we use for our empirical analysis starts close to the eruption of the global financial downturn, usually identified as occurring in late 2008, and extends over the following recession period. The crisis heavily affected Spanish exports, which faced a substantial drop in 2009, and was accompanied by an increase in unemployment rates throughout Spain. It is reasonable to expect that these peculiar dynamics have affected migrant composition, decreasing their incentives to stay in Spain and increasing those to expatriate, leading to a negative selection of the “stayers” and deteriorating their ability to facilitate trade. In Fig. 3, we provide an original way to address this issue. Specifically, we enrich the three-way fixed effects PPML regression with a full set of interaction effects between each of our migration variables and each year in our sample. Appendix Table 16 reports the estimated coefficients.

The coefficients display a clear pattern. In the case of immigrants, the pro-export effect is found to decrease and become insignificantly different from zero from 2010 onward. The pro-trade effect of emigrants is instead increasingly positive over time, and more markedly so since 2012. These results are important in at least two respects. On the one hand, they help reconcile our findings with those of previous studies and with the results by Peri and Requena-Silvente (2010) in particular. On the other hand, and relatedly, they suggest that a change in the composition of Spanish migrants may have occurred over time. This could have followed the stronger reaction to the crisis of more-qualified immigrants and emigrants, who are presumably better able to facilitate the creation of a trade tie.

Clearly, the fact that our panel starts in 2006 limits our ability to analyze the role of the Global Financial Crisis in the migrant pro-export effect. The main hindrance to this end is the lack of emigration data from before 2006. In Appendix B.3, we impute emigration data based on data on residential cancellations and study how migrant effects change across the pre- and post-crisis period. The results confirm that the global financial crisis reduced the immigrant effects and increased the emigrant effects.

In the same Appendix, we also study whether these dynamics could be attributed to asymmetric effects of immigration or return migration. We do not identify positive effects of return migration, but including this variable presumably reduces the noise in the estimates and makes the positive immigration effects gain significance at the 10% level.

## Conclusions

Although it represents a research issue with a long-standing tradition, the analysis of the pro-trade effects of migrants at the subnational level is still open to amelioration. In particular, recent advances in the gravity model literature offer a battery of new analytical tools with which previous knowledge about the quantity and quality of these effects can be proven and possibly enriched. This is primarily the case for the heterogeneity of regional multilateral resistance terms, in addition to region–country dyadic time-invariant fixed effects and time-varying country-level effects. While the inclusion of time-varying effects for the traders is an obvious implication of the gravity model, it has often been neglected in empirical studies on the migration–trade link that adopt subnational units. As we have argued, multiple motivations urge for the inclusion of these controls. As we have shown, their neglect entails serious distortions in the results. As we have also shown, additional important refinements can be obtained by taking stock of computational advances at the frontier of gravity model estimation via Pseudo Maximum Likelihood (PML) estimators with multi-way fixed effects. Through our diagnostic tests, implemented for the first time to the analysis of the migration–trade link and with panel data, we compared OLS, GPML, and PPML estimators. The Poisson PML (PPML) estimator emerged as the most suitable in the analysis of the pro-export effect of migrants for Spanish provinces. The magnitudes of the PPML, the Gamma PML (GPML), and the OLS estimates are comparable to each other, suggesting that the model is well specified and not substantially affected by the zeroes in the dependent variable. On the other hand, the OLS estimator was discarded on the grounds of heteroskedasticity, with important implications for the obtained results.

By exclusively relying on the OLS estimates, we would have concluded that immigrants exert a significant pro-trade effect along with emigrants, consistent with previous studies. Instead, neither of our PML models supports such an inference in the considered timeframe. Rather, our estimates indicate and robustly confirm a positive effect of emigrants only on the exports of Spanish provinces. This result is robust to an instrumental variables approach and to the bias correction proposed by Weidner and Zylkin (2020).

The magnitude of the effect identified implies that a 10% increase in the emigrant population from a given province to a certain country would increase its exports towards it by almost 1%. Disregarding the heterogeneous exporting capacity of provinces would have led to an undue overestimation of both immigrant and emigrant effects. When compared to the few previous studies that include both immigrants and emigrants in export effects, the stronger and more robust effect of emigrants represents an interesting result. These results could be interpreted in light of the peculiar phase of the business cycle and of the impact of the global financial downturn. These have apparently eroded immigrants’ abilities to promote trade. Omitting the outward side of migration would have made this distinction impossible and may have wrongly attributed to immigrants a role that is actually played by emigrants.

Importantly, the insignificant immigrant effects bear implications about the mechanism underlying the pro-trade effects. Indeed, the emigrant effect could be either an information, an enforcement, or a preference effect. Considering that no robust evidence could be found in support of immigrant effects, which are exclusively driven by the first two mechanisms, one may argue that the effects that we identify are due to a preference effect only. While this may be the primary mechanism driving emigrant effects, the results of our heterogeneity analysis still support immigrant effects compatible with the information effect: that is, immigration effects were found in high-concentration provinces and in provinces less specialized in intermediate goods. Accordingly, we are inclined to attribute the declining role of immigration to a deterioration in the trade opportunities that are amenable to the mediation of immigrants, as well as to a change in immigrant composition. Further research may seek to confirm this interpretation during more positive phases of the cycle and, more generally, may further investigate the relationship between the business cycle and the migration–trade link.

Original results also emerge when the new methodological setting that we have proposed is applied to investigate the nuances of the trade–migration link. Consistent with previous literature, the effect of emigration is stronger in the trade linkages that provinces establish with more institutionally distant countries, i.e., with non-EU countries in our setting. Institutional distance actually represents a transaction cost, which the business networks created by emigrants can contribute to alleviating. An opposite result emerges with respect to language commonality, as Spanish emigrants to Spanish- and English-speaking countries have a magnifying, rather than reduced, pro-export effect. This result suggests that language skills also directly affect the individual ability of migrants to promote exports. Hence, language commonality could be a leverage for better detecting and exploiting trade opportunities. In other words, among the potential trade opportunities that a migrant can facilitate, some could be lost due to language differences. This would have the effect of reducing the emigrants’ capacity to promote trade (cfr. the random encounter model in Wagner et al. 2002).

Additional insights from our application concern the novel contribution regarding the effect of networks of expatriates on exports via local and nation-wide networks. Results show that both matter. However, the existence of a network of Spanish expatriates in the same country is more important than their provinces of origin, with a very large elasticity. This likely indicates that an increase in emigrant stocks triggers a demand effect that not only promotes trade from the province of origin but also from Spain as a whole. The intertwining of local and non-local effects of emigration on trade also represents a newly emergent piece of evidence on which future research should concentrate.

## Figures and Tables

**Fig. 4:**
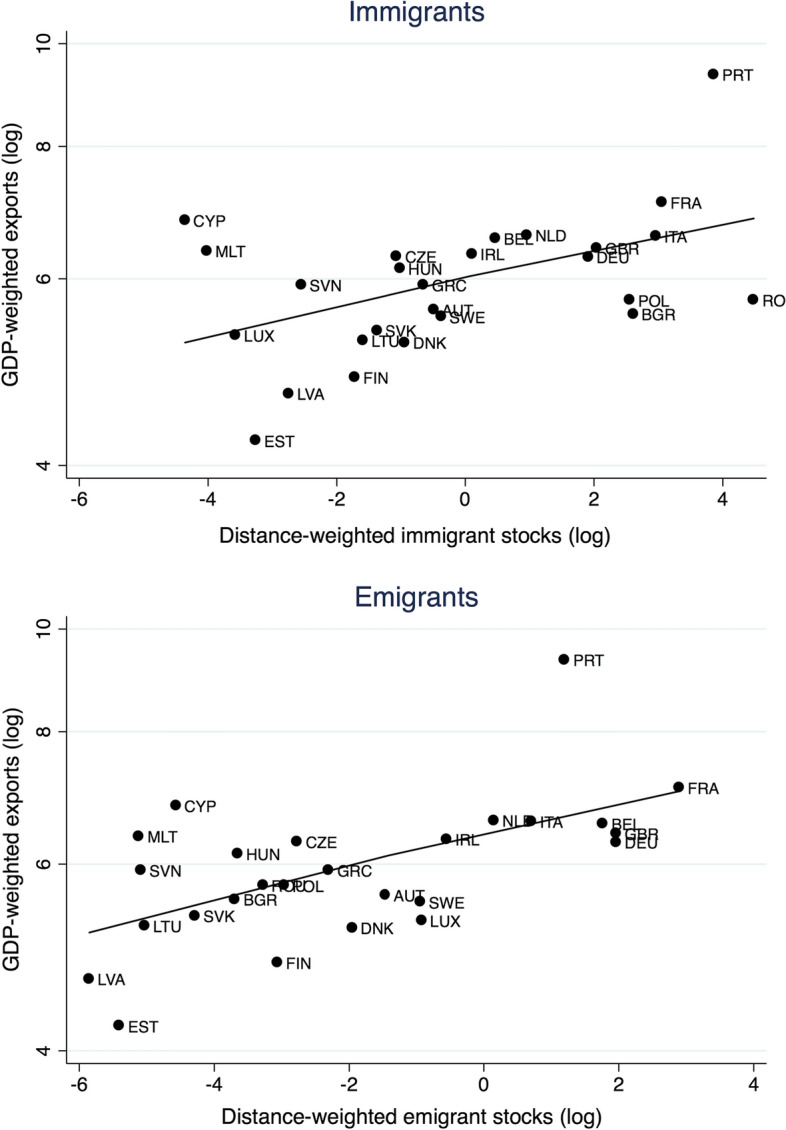
GDP-weighted exports towards EU countries vs. the respective distance-weighted immigrant and emigrant stocks: Madrid and EU countries, 2008. To mitigate the role of confounding factors, the figure only portrays EU countries. Similar results are obtained when looking at OECD countries. Our own elaboration of DataComex, INE, Censo Electoral de españoles residentes en el extranjero (CERA), and IMF data

**Fig. 5:**
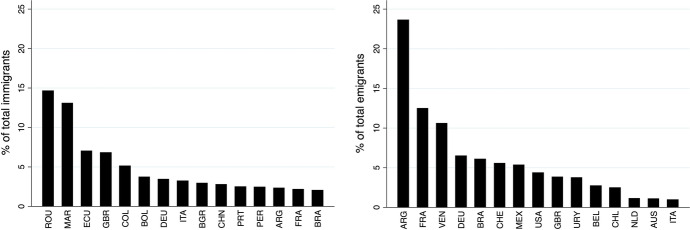
Top 15 origin and destination countries of immigrants and emigrants (2010). *Source* Own elaboration of INE data

## A Data appendix

We matched trade data with demographic data sourced from the Spanish National Statistical Institute (INE); our main variables and the relative sources are reported in Table 8. Some caveats are due concerning migration variables, whose characteristics (e.g., skills, employment status, and length of stay) are affected by severe data constraints at the subnational level. Drawing on the extant literature, we measure immigrants $Immi_{ijt}$ with the stock of residents registered in province *i* according to the municipal registries (“Padrón Municipal”) and who hold the citizenship of a country other than Spain *j* at time *t*.

As is well known in migration studies, this is an imperfect measure of immigration as it neglects the portion of foreign-born people that have acquired the nationality of the host country. Furthermore, the same stock refers only to formally residing people—it also neglects undocumented immigrants and intra-national movements of foreigners who are not registered in official changes of residence. Our measurement of emigrants, $Emi_{ijt}$, captured (as in Flisi and Murat 2011) by the stock of people recorded in the Spanish election registries of province *i* who have moved their residence to foreign country *j* at time *t* is also imperfect. Our variable likely underestimates the actual emigrant stocks. Indeed, migrants typically register in the electoral registries when they are fairly established in a foreign country and intend their stay to be long-term. Hence, we may underestimate the emigrants of more recent expatriation. Furthermore, while the data report the emigrants’ last province of residence in Spain, they are uninformative about the emigrants’ country of birth, such that we cannot distinguish return migrants from native Spanish expatriates. Yet, maintaining one’s voting rights in Spain implies the persistence of strong ties to Spain. Thus, it seems to us safe to assume that $Emi_{ijt}$ reflects the dynamics of the Spanish emigrant population more than the dynamics of return migration.

Table 5 reports the summary statistics for the main variables of our application. The inspection of the summary statistics reveals that the distribution of our dependent variable is characterized by a right skew and many small values. This is typical of trade data, and even more so of trade data on subnational units, which typically display more variation for province–country pairs characterized by larger trade volumes. This is an indication of heteroskedasticity, which we address in our empirical strategy. As for the issue of zero trade flows, 7.45% of our trade observations have a nil value. The shares of zeroes in our variables of interest ($Immi_{ijt}$ and $Emi_{ijt}$) are 8% and 28%, respectively.

Table 6 displays the correlation matrix. First of all, note that the correlations between exports ($X_{ijt}$) and each of the two migration variables (0.182 with $Immi_{ijt}$ and 0.281 with $Emi_{ijt}$) are higher than that between the two. The correlation between $Immi_{ijt}$ and $Emi_{ijt}$ is actually quite low (0.115). Furthermore, the main origin and destination countries of immigrants and emigrants and their respective distributions differ substantially (see Fig. 5 for the year 2010). The distribution patterns of immigrants and emigrants by Spanish province are also quite distinct (still for 2010, see Table 7 in the Appendix). As argued in Sect. 2.1, $Immi_{ijt}$ and $Emi_{ijt}$ can be assumed to portray different phenomena and, supporting the first of our methodological choices, are both in need of consideration.

A descriptive overview of the data suggests that both immigration and emigration can have an impact on exports at the province level. For illustration, Fig. 4 focuses on the province of Madrid in 2010 and plots the exports-to-GDP ratio against the immigrant and emigrant stocks, weighted by distance, for each OECD partner country. The relationship appears positive for both immigration and emigration, and slightly stronger for the former than for the latter. The province of Madrid exports more to countries where immigrants and emigrants have helped build up larger transnational communities.

**Table 5:** Summary statistics

Variable	Mean	Std. Dev.	Min	Max
Exports $X_{ijt}$	36557.36	202660.2	0	7718364
Prov. p.c. gross product $Y_{it}$	21.56469	4.512604	14.63577	38.10292
Country p.c. GDP $Y_{jt}$	16.1169	21.09531	.2492031	120.7873
Immigrants $Immi_{ijt}$	954.8313	5459.894	0	219567
Emigrants $Emi_{ijt}$	266.0714	1692.895	0	55022
No immigrant $NI_{ijt}$	.0766267		0	1
No emigrant $NE_{ijt}$	.2846636		0	1
Km distance $Dist_{ij}$	4985.11	3487.489	164.6923	19959.6

Observations: 54,200

**Table 6:** Correlations

Variable		1	2	3	4	5	6	7	8
1	$X_{ijt}$	1.0000							
2	$Y_{it}$	0.1194	1.0000						
3	$Y_{jt}$	0.1543	$-$0.0044	1.0000					
4	$Immi_{ijt}$	0.1737	0.0802	$-$0.0150	1.0000				
5	$Emi_{ijt}$	0.2743	0.0271	0.0810	0.1065	1.0000			
6	$NI_{ijt}$	$-$0.0494	$-$0.0451	0.0353	$-$0.0505	$-$0.0447	1.0000		
7	$NE_{ijt}$	$-$0.1094	$-$0.0555	$-$0.3006	$-$0.0974	$-$0.0996	0.1952	1.0000	
8	$Dist_{ij}$	$-$0.1193	$-$0.0134	$-$0.1676	$-$0.0349	0.0567	$-$0.0032	$-$0.1616	1.0000

**Table 7:** Total population, immigrants, and emigrants by province (2010)

Province	Population	Nationality		Prov. pop.		Imm. pop.	Emigrants	Prov. pop.		Emi. pop.
	(Persons)	Spanish	Foreigners	%	Level	%		%	Level	%
SPAIN	47,021,031	41,273,297	5,747,734	12.2	–	100	1,408,825	3.0	–	100
Alicante	1,926,285	1,459,186	467,099	24.2	High	8.1	21,371	1.1	Low	1.5
Baleares	1,106,049	863,793	242,256	21.9	High	4.2	14,328	1.3	Low	1.0
Almería	695,560	544,401	151,159	21.7	High	2.6	27,772	4.0	High	2.0
Girona	753,046	590,799	162,247	21.5	High	2.8	9884	1.3	Low	0.7
Tarragona	808,420	658,106	150,314	18.6	High	2.6	10,087	1.2	Low	0.7
Castellón	604,274	492,009	112,265	18.6	High	2.0	5,267	0.9	Low	0.4
Lleida	439,768	359,278	80,490	18.3	High	1.4	11,471	2.6	Mid	0.8
Málaga	1,609,557	1,334,530	275,027	17.1	High	4.8	33,211	2.1	Mid	2.4
Madrid	6,458,684	5,378,740	1,079,944	16.7	High	18.8	174,819	2.7	Mid	12.4
Murcia	1,461,979	1,220,114	241,865	16.5	High	4.2	19,607	1.3	Mid	1.4
Guadalajara	251,563	212,359	39,204	15.6	High	0.7	2247	0.9	Low	0.2
S.C.Tenerife	1,027,914	874,587	153,327	14.9	High	2.7	72,454	7.0	High	5.1
Barcelona	5,511,147	4,705,660	805,487	14.6	High	14.0	104,302	1.9	Mid	7.4
La Rioja	322,415	275,735	46,680	14.5	High	0.8	10,237	3.2	Mid	0.7
Las Palmas	1,090,605	936,553	154,052	14.1	Mid	2.7	25,548	2.3	Mid	1.8
Zaragoza	973,252	845,610	127,642	13.1	Mid	2.2	15,388	1.6	Mid	1.1
Cuenca	217,716	189,747	27,969	12.8	Mid	0.5	2269	1.0	Low	0.2
Segovia	164,268	143,194	21,074	12.8	Mid	0.4	2304	1.4	Mid	0.2
Valencia	2,581,147	2,266,752	314,395	12.2	Mid	5.5	36,944	1.4	Mid	2.6
Huesca	228,566	200,756	27,810	12.2	Mid	0.5	5063	2.2	Mid	0.4
Teruel	145,277	127,643	17,634	12.1	Mid	0.3	3656	2.5	Mid	0.3
Toledo	697,959	613,984	83,975	12.0	Mid	1.5	6627	0.9	Low	0.5
Melilla	76,034	67,161	8873	11.7	Mid	0.2	3527	4.6	High	0.3
Navarra	636,924	565,555	71,369	11.2	Mid	1.2	16,766	2.6	Mid	1.2
Soria	95,258	85,388	9870	10.4	Mid	0.2	4421	4.6	High	0.3
Burgos	374,826	340,260	34,566	9.2	Mid	0.6	12,122	3.2	Mid	0.9
Araba/Álava	317,352	289,142	28,210	8.9	Mid	0.5	4139	1.3	Low	0.3
C. Real	529,453	483,452	46,001	8.7	Mid	0.8	4175	0.8	Low	0.3
Huelva	518,081	475,328	42,753	8.3	Mid	0.7	5200	1.0	Low	0.4
Albacete	401,682	369,277	32,405	8.1	Mid	0.6	5129	1.3	Low	0.4
Ávila	171,896	159,283	12,613	7.3	Mid	0.2	6005	3.5	Mid	0.4
Granada	918,072	853,738	64,334	7.0	Mid	1.1	34,317	3.7	High	2.4
Cantabria	592,250	553,049	39,201	6.6	Mid	0.7	25,170	4.2	High	1.8
Valladolid	533,640	500,984	32,656	6.1	Mid	0.6	9005	1.7	Mid	0.6
Gipuzkoa	707,263	664,814	42,449	6.0	Mid	0.7	19,313	2.7	Mid	1.4
Bizkaia	1,153,724	1,085,014	68,710	6.0	Mid	1.2	27,011	2.3	Mid	1.9
León	499,284	473,321	25,963	5.2	Mid	0.5	35,339	7.1	High	2.5
Ourense	335,219	318,508	16,711	5.0	Mid	0.3	82,134	24.5	High	5.8
Ceuta	80,579	76584	3995	5.0	Low	0.1	2132	2.6	Mid	0.2
Salamanca	353,619	336,113	17,506	5.0	Low	0.3	23,265	6.6	High	1.7
Asturias	1,084,341	1,035,055	49,286	4.5	Low	0.9	83,041	7.7	High	5.9
Palencia	172,510	165,301	7209	4.2	Low	0.1	5510	3.2	Mid	0.4
Zamora	194,214	186,173	8041	4.1	Low	0.1	14,820	7.6	High	1.1
Pontevedra	962,472	922,678	39,794	4.1	Low	0.7	106,279	11.0	High	7.5
Sevilla	1,917,097	1,840,007	77,090	4.0	Low	1.3	22,326	1.2	Low	1.6
Lugo	353,504	339,328	14,176	4.0	Low	0.2	50,352	14.2	High	3.6
Cédiz	1,236,739	1,188,972	47,767	3.9	Low	0.8	19,825	1.6	Mid	1.4
Cáceres	415,083	399,767	15,316	3.7	Low	0.3	12,705	3.1	Mid	0.9
Badajoz	692,137	668,097	24,040	3.5	Low	0.4	8803	1.3	Low	0.6
A Coruña	1,146,458	1,107,469	38,989	3.4	Low	0.7	128,090	11.2	High	9.1
Córdoba	805,108	779,849	25,259	3.1	Low	0.4	13,920	1.7	Mid	1.0
Jaén	670,761	650,094	20,667	3.1	Low	0.4	9128	1.4	Mid	0.6

**Table 8:** Main Data Sources

Var.	Description	Source
$$X_{ijt}$$	Value of the exports from province *i* to country *j* in year *t* (thousands of €)	Datacomex, http://datacomex.comercio.es/principal_comex_es.aspx. Full database at 3-digit disaggregation requested and received by email.
$$Y_{jt}$$	GDP of country *j* in year *t* (billions of US$)	IMF (International Monetary Fund) World Economic Outlook Database, http://www.imf.org/external/pubs/ft/weo/2012/01/weodata/index.aspx
$$Y_{it}$$	Gross product of province *i* in year *t* (thousands of €)	INE (*Instituto Nacional de Estadística*) - “PIB a precios de Mercado precios Corrientes”, http://www.ine.es/jaxi/menu.do?L=0&type=pcaxis
$${\text {Immi}}_{ijt}$$, $${\text {NI}}_{ijt}$$	Foreign residents with country *j* nationality residing in province *i* in year *t*	INE - “Población extranjera por sexo, comunidades y provincias y nacionalidad” http://www.ine.es
$${\text {Emi}}_{ijt}$$, $${\text {NE}}_{ijt}$$	Spanish expatriates registered in province *i* and residing in country *j* in year *t*	Censo Electoral de españoles residentes en el extranjero (CERA) por provincia de inscripción y país de residencia, http://www.ine.es/ss/Satellite?c=Page&cid=1254735793323
$${\text {Dist}}_{ij}$$	Great-circle distance between the main center of province *i* and the capital of country *j*	Google Maps

## B Robustness checks

### B.1 Results excluding distance

**Table 9:** Baseline results excluding distance

	(1)	(2)	(3)	(4)	(5)	(6)	(7)	(8)	(9)
	PPML			OLS			GPML		
$${\text {In}}({\text {Immi}}_{jit-1}+1)$$	0.079$$**$$		0.053	0.060$$***$$		0.048$$**$$	0.034$$*$$		0.021
	(0.038)		(0.037)	(0.022)		(0.022)	(0.019)		(0.019)
${\text {NI}}_{jit-1}$	$-$0.102		$-$0.133	0.050		0.026	$-$0.018		$-$0.045
	(0.092)		(0.091)	(0.068)		(0.068)	(0.054)		(0.054)
$${\text {In}}({\text {Emi}}_{jit-1}+1)$$		0.115$$***$$	0.095$$**$$		0.105$$***$$	0.095$$***$$		0.104$$***$$	0.101$$***$$
		(0.040)	(0.038)		(0.026)	(0.027)		(0.023)	(0.023)
${\text {NE}}_{jit-1}$		0.005	$-$0.001		0.047	0.041		0.068$*$	0.066$*$
		(0.068)	(0.068)		(0.043)	(0.043)		(0.036)	(0.036)
N	54,250	54,250	54,250	50,210	50,210	50,210	50,210	50,210	50,210
Province-time effects	Yes	Yes	Yes	Yes	Yes	Yes	Yes	Yes	Yes
Country-region effects	Yes	Yes	Yes	no	Yes	Yes	Yes	Yes	Yes
Country-time effects	Yes	Yes	Yes	Yes	Yes	Yes	Yes	Yes	Yes

Standard errors in parentheses

$$*$$$$p<0.1$$, $$**$$$$p<0.05$$, $$***$$$$p<0.01$$

### B.2 Do subnational heterogeneous MRTs matter?

As we claimed in Sect. 2.2, allowing for heterogeneous export capacities across provinces is recommendable for different reasons. Yet, given the relatively small size of Spanish provinces, one may question whether this theoretically founded yet quite demanding approach yields statistically different implications from ones that assume the export capacity to be homogeneous country-wide or within the same NUTS2 region. In order to address this issue, we compare our previous results with those of two alternative specifications (Table 10).

In the first (columns 1–3), subnational MRTs are assumed equal across provinces pertaining to the same region, similarly to Bratti et al. (2014). In a second specification (columns 4–6), any subnational heterogeneity in the exporting capacity is ruled out, while region-country FEs and country-year FEs are still included.

As expected, the assumption of homogeneous export capacities among the provinces of the same region (columns 1–3) strongly overestimates the effects of both our focal variables.^28^ Across the three estimators, both the immigration and emigration coefficients increase substantially. This confirms our expectation of correlation between bilateral stocks of migrants and the exporting capacities of provinces, which are omitted when the subregional heterogeneity is assumed out. As mentioned in Sect. 2.2, most of the exporting capacity as well as most of immigrant and emigrant stocks are concentrated in provinces like Madrid, Barcelona, and Valencia. Allowing for heterogeneous exporting capacities at the relatively aggregated NUTS2 level may, in fact, confound these effects. This interpretation is supported when we look at the coefficient of the distance variable, which turns out to be implausibly positive and significant in these estimates. In Spain, the regional capitals are often located at the geographic center of their respective regions, and they are thus, on average, more distant to any trading partner. If we do not control for the exporting capacity of provinces, the effects of the location of the largest exporters and their exporting capacity will get mistakenly conflated in the coefficient of distance.^29^ We actually view this result as a demonstration of the correctness of our preferred approach. When controlling for province-level exporting capacity, indeed, the effect becomes insignificant (PPML estimates) or negative (OLS and GPML estimates) and, as mentioned, the results are almost insensitive to whether distance is included or not.

**Table 10:** Pro-export effect of immigrants and emigrants: alternative FE specifications

	(1)	(2)	(3)	(4)	(5)	(6)
	PPML	OLS	GPML	PPML	OLS	GPML
$${\text {In}}(Y_{jt-1} \times Y_{it-1})$$	5.730$$***$$	7.182$$***$$	6.331$$***$$	5.389$$***$$	6.866$$***$$	5.958$$***$$
	(0.375)	(0.230)	(0.192)	(0.360)	(0.223)	(0.189)
$${\text {In}}({\text {Immi}}_{ijt-1}+1)$$	0.194$$***$$	0.323$$***$$	0.275$$***$$	0.191$$***$$	0.322$$***$$	0.277$$***$$
	(0.050)	(0.023)	(0.017)	(0.050)	(0.023)	(0.017)
$${\text {In}}({\text {Emi}}_{ijt-1}+1)$$	0.639$$***$$	0.615$$***$$	0.571$$***$$	0.636$$***$$	0.614$$***$$	0.565$$***$$
	(0.046)	(0.028)	(0.024)	(0.046)	(0.028)	(0.024)
$${\text {NI}}_{ijt-1}$$	-0.271$$**$$	$$-$$0.224$$***$$	$$-$$0.132$$**$$	$$-$$0.265$$**$$	$$-$$0.227$$***$$	$$-$$0.134$$**$$
	(0.117)	(0.078)	(0.062)	(0.117)	(0.078)	(0.062)
$${\text {NE}}_{ijt-1}$$	0.267$$***$$	0.160$$***$$	0.203$$***$$	0.248$$***$$	0.154$$***$$	0.186$$***$$
	(0.081)	(0.053)	(0.044)	(0.080)	(0.053)	(0.045)
$${\text {In}}({\text {Dist}}_{ij})$$	1.170$$**$$	0.158	1.181$$***$$	1.128$$**$$	0.089	1.114$$***$$
	(0.460)	(0.422)	(0.397)	(0.456)	(0.419)	(0.393)
N	54,250	50,210	50,210	54,250	50,210	50,210
Province-year effects	No	No	No	No	No	No
Country-year effects	Yes	Yes	Yes	Yes	Yes	Yes
Region-country effects	Yes	Yes	Yes	Yes	Yes	Yes
Region-year effects	Yes	Yes	Yes	No	No	No

Standard errors clustered at the pair level in parentheses

$$*$$$$p<0.1$$, $$**$$$$p<0.05$$, $$***$$$$p<0.01$$

We obtain similar results when we exclude region-time effects altogether (columns 4–6), which corresponds to an assumption of homogeneous exporting capacity throughout Spain. Somewhat surprisingly, the results are similar to those in columns 1–3. This suggests that the main source of heterogeneity is actually to be found at a quite refined geographic level, like the NUTS3 level we are considering. Once the province-year effects are omitted, including region-time effects does not seem to substantially affect the results.

In conclusion, allowing for subnational heterogeneous MRTs actually does make a difference in the appreciation of trade effects of migration. In particular, assuming homogeneous MRTs leads to a pro-export effect of emigrants between almost 7 and almost 9 times greater.^30^

### B.3 Migration effects over the global financial crisis and return migration

In Sect. 4.6, we identify a declining pattern in the effects of immigration and an increasing pattern for emigration. To gain more insight on the role of the Global Financial Crisis in this regard, in this section we address the lack of emigration data before 2006.

INE official statistics on emigration start in 2006, not only at the province–country level but even at the national level. The same applies to Eurostat data. Emigration data from other sources (e.g., the OECD DIOC database) are not available on a yearly basis. Moreover, given the marked changes in the immigration dynamics that were set off by the Global Financial Crisis, an extrapolation of the available data to the pre-crisis years (for which we have immigration data), may be misleading. Hence, we address the issue drawing on the availability of the same data on residential inscription cancellations that we used to construct our emigration instrument (see Sect. 3.1.3). This approach should allow us to mitigate the possible distortions in the imputation process that may derive from extrapolating crisis-period data to pre-crisis years. Specifically, we impute pre-crisis emigration data based on a set of PPML fixed effects regressions. The bilateral stocks of emigrants $Emi_{ijt}$ are regressed on the flows of Spanish emigrants from province *i* to any country in year *t*; the flows of Spanish emigrants from any province to country *j* in year *t*; the interaction of the first two regressors; and a continuous time variable. The predicted value of the emigration stocks, rounded to the nearest integer, is used to impute the pre-2006 values of emigration. Due to the fact that data on the residential cancellations only go back to 2002, the length of the resulting panel is 14 years, i.e., 2002–2016. As a robustness check, we compare the results of this imputation procedure with those of a more standard extrapolation of the emigration data to the pre-crisis period.

We implement our preferred specification over this longer time period to study the robustness of the overall results and then add a set of mutually exclusive interaction effects of all regressors with the pre-crisis period—we do not see an obvious indicator for the end of the crisis period, given the persistently high levels of unemployment.

The results are reported in Table 11, with columns (3)–(4) displaying the results for our preferred imputation procedure (“Imputation Strategy 1”) and columns (5)–(6) those stemming from a standard extrapolation (“Imputation Strategy 2”). On the whole, the results for the longer time period are consistent with the main findings of the paper. As for the interaction with the crisis, the results indicate a pattern that is consistent across imputation procedures and further reconciles our results with those of the previous literature: in the pre-crisis period, immigration exerted a positive and significant effect on exports, while emigration was positive but insignificant. With the crisis, the inference switches: emigrants become positive and significant and immigrants positive and insignificant. These results suggest that the economic conjuncture and compositional effects play a role in the pro-trade effects of immigrants and emigrants.

In this regard, it is important to recognize that an implicit assumption of our model is that the effects of an increase in the migration stocks are symmetric to those of a decrease. This may not be the case if immigrants returning to their home countries maintain ties to Spain and still promote the realization of bilateral trade opportunities. In this case, we would expect a negative effect of immigration: the decrease in immigrant stocks, indicating a return to their home countries, would increase bilateral trade. This negative effect of declining stocks could potentially offset the positive effects of growing stocks and explain the declining and relatively small aggregate coefficient of immigration that we observe.

To address this issue, we run a number of checks including interaction effects with indicator variables for the negative growth of immigration stocks, but we do not find any evidence supporting the presence of asymmetric effects, at least in our data.^31^

Still, it may be that we do not identify an asymmetry in the immigrant effects due to inappropriate data. Indeed, we only observe a positive or negative change in the stocks of immigrants, but we cannot tell whether a negative change is due to a return to the home country, a transfer to a third destination country, or even to a negative demographic balance or a transfer to a different Spanish province. In turn, for the reasons detailed in Appendix A, the election-related nature of our emigration variable is unlikely to be capturing return migration. This implies that even if an asymmetric effect of immigration for increases and decreases in the migration stocks is plausible, it is unlikely to be captured in our immigration data.

Drawing on microdata on residential cancellations (available from the INE upon request), we identify the subset of residential cancellations that are likely to be motivated by return migration. Specifically, we consider as return migration the changes of residence stemming from Spanish provinces by foreign-born persons that target foreign countries whenever (i) the person is moving residence towards the country of her nationality; or (ii) the person is moving residence towards her country of birth. In 51% of the identified cases of return, the nationality coincides with the country of birth. The data provide the necessary level of detail from 2006 onward.

Before 2006, dyadic yearly data on return migration are unavailable, but INE provides data on residential cancellations aggregated by whether the request was filed by a Spanish or a foreign national and detail them by either province of cancellation, country of nationality, or country of destination. The combined probability that a random foreign national who leaves Spain in year *t* is a return migrant going back to her origin country *j* from province *i* can be proxied as the combined probability that this foreign person is coming from province *i* and is targeting country *j*.^32^ We approximate these probabilities based on the relative frequencies of residential cancellations and multiply them by the number of foreign nationals leaving the country to get an estimated pre-2006 flow of return migrants.

To approximate the stocks of return migrants, we cumulate the estimated flows. Finally, we take logs adding one unit to the stocks to make the measure comparable with those of immigration and emigration. If ties persist between the previous immigration destination and the origin countries after return, we would expect that return migration has a positive effect on trade.

In panel (b) of Table 11, we report the results of a set of specifications that include our measure of return migration. When we pool our estimates over the entire period (either over 2007–2016 in column 1 or over 2002–2016 in columns 3 and 5), the coefficient of return migrants is negative and insignificant. Interestingly, however, the inclusion of this variable increases the positive coefficient of immigration and makes it significant at the 10% level.

When we split the coefficients into their before-crisis and crisis periods (columns 2, 4, and 6), the estimates consistently reveal a negative effect of return migrants on trade before 2008 that attenuates during the crisis times. Hence, there does not seem to be evidence of a positive effect of return migrants. Rather, the decrease in immigration stocks associated with return decreases trade, indicating that the smaller stocks effectively decrease the probability that immigrants promote exports. In other words, we interpret the estimates in panel (b) as a suggestion that the effect is indeed symmetric. Nonetheless, the decrease in the negative effect of return migrants observed during the crisis may be seen as further evidence that the composition of immigrants plays a role in exerting pro-trade effects.

**Table 11:** Pre-/post 2008 and return migration

Period	Baseline		Imputation strategy 1		Imputation strategy 2	
	2007–2016		2002–2016		2002–2016	
	(1)	(2)	(3)	(4)	(5)	(6)
**Panel (a)**						
${\text {In}}({\text {Immi}}_{ijt-1}+1)$	0.056		0.051		0.057$*$	
	(0.036)		(0.033)		(0.033)	
$${\text {In}}({\text {Emi}}_{ijt-1}+1)$$	0.095$$**$$		0.084$$**$$		0.055$$**$$	
	(0.037)		(0.038)		(0.026)	
$${\text {In}}({\text {Immi}}_{ijt-1}+1)\times ({\text {year}} <2008)$$		0.076$$**$$		0.070$$**$$		0.075$$**$$
		(0.036)		(0.033)		(0.032)
${\text {In}}({\text {Immi}}_{ijt-1}+1)\times ({\text {year}} \ge 2008)$		0.054		0.046		0.047
		(0.037)		(0.035)		(0.035)
${\text {In}}({\text {Emi}}_{ijt-1}+1)\times ({\text {year}} <2008)$		0.049		0.040		0.017
		(0.038)		(0.042)		(0.022)
$${\text {In}}({\text {Emi}}_{ijt-1}+1)\times ({\text {year}} \ge 2008)$$		0.101$$***$$		0.097$$***$$		0.090$$***$$
		(0.038)		(0.038)		(0.033)
**Panel (b)**						
${\text {In}}({\text {Immi}}_{ijt-1}+1)$	0.065*		0.061*		0.067**	
	(0.034)		(0.031)		(0.031)	
${\text {In}}({\text {Emi}}_{ijt-1}+1)$	0.099***		0.089**		0.059**	
	(0.037)		(0.038)		(0.026)	
${\text {In}}({\text {Return}}_{ijt-1}+1)$	$-$0.019		$-$0.026		$-$0.025	
	(0.035)		(0.020)		(0.020)	
${\text {In}}({\text {Immi}}_{ijt-1}+1)\times ({\text {year}} <2008)$		0.087**		0.073**		0.078**
		(0.036)		(0.033)		(0.033)
${\text {In}}({\text {Immi}}_{ijt-1}+1)\times ({\text {year}} \ge 2008)$		0.062*		0.054		0.054*
		(0.035)		(0.033)		(0.033)
${\text {In}}({\text {Emi}}_{ijt-1}+1)\times ({\text {year}} <2008)$		0.052		0.041		0.022
		(0.038)		(0.041)		(0.022)
${\text {In}}({\text {Emi}}_{ijt-1}+1)\times ({\text {year}} \ge 2008)$		0.105***		0.100***		0.094***
		(0.038)		(0.038)		(0.032)
${\text {In}}({\text {Return}}_{ijt-1}+1)\times ({\text {year}} <2008)$		$-$0.045		$-$0.059***		$-$0.061***
		(0.036)		(0.017)		(0.018)
${\text {In}}({\text {Return}}_{ijt-1}+1)\times ({\text {year}} \ge 2008)$		$-$0.017		$-$0.015		$-$0.015
		(0.039)		(0.033)		(0.033)
N	54,250	54,250	77,734	77,734	80,900	80,900

PPML estimates. Standard errors clustered at the province-country level in parentheses.

In Columns (3)–(4) emigrants are imputed based on our preferred imputation procedure

that draws on residential inscription cancellations data (see text).

In Columns (5)–(6), emigrants are imputed based on a standard extrapolation.

$$*$$$$p<0.1$$, $$**$$$$p<0.05$$, $$***$$$$p<0.01$$

## C Heterogeneity

### C.1 Institutional similarity and language commonality

Previous studies Girma and Yu (2002); Dunlevy (2006) have argued that migrant effects are larger with greater institutional distance between partners. Briant et al. (2014) argue that the informal enforcement mechanisms operating within social and business networks are more effective when the risk for predatory behavior is greater. This may be the case and may be due to, for example, substantial differences in the quality of the arbitration tribunals or in the rules setting the standards for product quality. Conversely, institutional similarity, by reducing transaction costs, reduces the saliency of the migrants’ pro-trade effects (Peri and Requena-Silvente 2010).

The literature has applied similar considerations to the role of language commonality (Wagner et al. 2002; Dunlevy 2006; Briant et al. 2014), arguing that the lack of a common linguistic and cultural background makes the role of migrants more significant in reducing the transaction and fixed costs of trade.

To test the previous arguments in our setting, in the following we include interaction terms between our focal variables, ${\text {In}}(Immi_{ijt})$ and ${\text {In}}(Emi_{ijt})$, and our proxies for institutional and language similarity. For the former, we use a dummy for whether the partner country is an EU member (*EU*) and for the latter, a dummy identifying whether Spanish is an official language ($Spanish-Speaking$). To facilitate the interpretation of interaction terms in Poisson models, we include the interaction terms of ${\text {In}}(Immi_{ijt})$ and ${\text {In}}(Emi_{ijt})$ with the relevant dummy and with its complement to one (respectively, *NEU* and $non-Spanish-Speaking$; this is in line with Girma and Yu 2002).

In what follows, we report the results of the PPML estimates for brevity, but the results are generally robust to the use of different estimators.

As for institutional similarity, column (1) of Table 12 shows that, consistent with previous literature, the pro-export effect of emigrants is unambiguously driven by emigrants towards extra-EU countries.

As for language commonality, instead, column (2) of Table 12 shows that emigrants residing in Spanish-speaking countries increase exports about twice as much as emigrants towards non-Spanish speaking ones. The difference is, however, not significant. Notice that this positive pro-export effect of Spanish-speaking emigrants is additional to the positive role of language commonality between trade partners, which is captured by the fixed effects of the model.

The above results may raise the question of whether they are attributable to the language effect per se or, rather, to the presence of remote colonial ties between Spain and the partner countries—hence, to another form of institutional similarity. The two effects are, indeed, empirically indistinguishable, given that all Spanish-speaking countries in our data (Latin American countries, the Philippines, and Equatorial Guinea) are former Spanish colonies.

To get further insight on this issue, we perform a set of additional checks that are reported in columns (3–5). In column (3) we implement a similar specification to that in column (2), where we further add a dummy variable, “Romance/Not-Spanish”, equal to one if one of the official languages of the partner country belongs to the Romance family but is not Spanish (this is the case for Italian, French, Romanian, and Portuguese), according to the classification provided by Ethnologue, www.ethnologue.com (cfr. Guiso et al. 2009). Note that this includes a number of former French and Portuguese colonies who have French as their official language. The results indicate no significant effects of emigration towards countries speaking languages in the Romance family but are otherwise very similar to the ones in column (2).

In column (4), we aggregate all languages of the Romance family into one group, “Romance”, and re-run our estimates including the mutually exclusive interaction effects between this variable and migration. While confirming the significant positive effects of emigration on trade, the results do not indicate significant differences between the effects of emigrants who target Romance-family countries and those who do not.

The above results could be interpreted in two different ways: first, what matters for emigrant trade promotion effects is not so much language similarity but rather language commonality (i.e., that they speak the language of the destination country and not just a similar one); or second, what matters for emigrant trade promotion effects is colonial ties (and, consequently, institutional proximity other than EU Membership). To gain further insight on this, we take a somewhat novel approach. We recognize that the vast majority of Spanish students learn English as a foreign language (in 2017, this share was as high as 95% according to Eurostat). If the language commonality argument prevails over the colonial ties argument, we should observe additional trade-promotion effects of emigrants towards English-speaking countries. To this end, we construct an additional dummy, “Spa+Eng”, equal to one if the partner country has either Spanish or English as an official language. As before, we construct a set of mutually exclusive interaction terms between this variable and our migration variables. The results, reported in column (5), support the language commonality interpretation, indicating a much stronger effect of emigrants towards Spanish- or English-speaking countries than other countries (furthermore, according to CEPII Chelem data^33^, the US features a share of Spanish-speaking residents that exceeds 9%; in this case, the effects of the Spanish and English languages probably add to each other). Overall then, the results provide more support to the interpretation of the magnifying effects of language commonality, rather than of colonial ties.

**Table 12:** Heterogeneity by institutional and language similarity

EU membership		Spanish-speaking		Spanish/Romance family		Romance family		Spanish/English	
	(1)		(2)		(3)		(4)		(5)
${\text {In}}({\text {Immi}}_{ijt-1}^{EU})$	0.061	${\text {In}}({\text {Immi}}_{ijt-1}^{Spa})$	0.125	${\text {In}}({\text {Immi}}_{ijt-1}^{Spa})$	0.120	${\text {In}}({\text {Immi}}_{ijt-1}^{Romance})$	0.057	${\text {In}}({\text {Immi}}_{ijt-1}^{Spa+Eng})$	-0.003
	(0.045)		(0.103)		(0.103)		(0.060)		(0.065)
${\text {In}}({\text {Immi}}_{ijt-1}^{non-EU})$	0.029	${\text {In}}({\text {Immi}}_{ijt-1}^{non-Spa})$	0.048	${\text {In}}({\text {Immi}}_{ijt-1}^{non-Romance})$	0.039	${\text {In}}({\text {Immi}}_{ijt-1}^{non-Romance})$	0.041	${\text {In}}({\text {Immi}}_{ijt-1}^{non-Spa+Eng})$	0.045
	(0.040)		(0.037)		(0.039)		(0.039)		(0.041)
				${\text {In}}({\text {Immi}}_{ijt-1}^{Romance/Non-Spa})$	0.047				
					(0.062)				
$${\text {In}}({\text {Emi}}_{ijt-1}^{EU})$$	0.032	$${\text {In}}({\text {Emi}}_{ijt-1}^{Spa})$$	0.163$$*$$	$${\text {In}}({\text {Emi}}_{ijt-1}^{Spa})$$	0.161$$*$$	$${\text {In}}({\text {Emi}}_{ijt-1}^{Romance})$$	0.093$$**$$	$${\text {In}}({\text {Emi}}_{ijt-1}^{Spa+Eng})$$	0.225$$***$$
	(0.045)		(0.089)		(0.088)		(0.047)		(0.071)
$${\text {In}}({\text {Emi}}_{ijt-1}^{non-EU})$$	0.168$$***$$	$${\text {In}}({\text {Emi}}_{ijt-1}^{non-Spa})$$	0.087$$**$$	$${\text {In}}({\text {Emi}}_{ijt-1}^{non-Romance})$$	0.090$$**$$	$${\text {In}}({\text {Emi}}_{ijt-1}^{non-Romance})$$	0.095$$**$$	$${\text {In}}({\text {Emi}}_{ijt-1}^{non-Spa+Eng})$$	0.058
	(0.043)		(0.039)		(0.045)		(0.046)		(0.037)
				${\text {In}}({\text {Emi}}_{ijt-1}^{Romance/Non-Spa})$	0.075				
					(0.049)				
${\text {NI}}_{ijt-1}$	-0.117	${\text {NI}}_{ijt-1}$	-0.141	${\text {NI}}_{ijt-1}$	-0.130	${\text {NI}}_{ijt-1}$	-0.130	${\text {NI}}_{ijt-1}$	-0.134
	(0.090)		(0.092)		(0.095)		(0.095)		(0.095)
${\text {NE}}_{ijt-1}$	0.055	${\text {NE}}_{ijt-1}$	-0.013	${\text {NE}}_{ijt-1}$	0.008	${\text {NE}}_{ijt-1}$	0.011	${\text {NE}}_{ijt-1}$	-0.017
	(0.071)		(0.068)		(0.069)		(0.069)		(0.065)
${\text {In}}({\text {Dist}}_{ij})$	-0.022	${\text {In}}( {\text {Dist}}_{ij})$	0.002	${\text {In}}({\text {Dist}}_{ij})$	-0.034	${\text {In}}({\text {Dist}}_{ij})$	-0.002	${\text {In}}({\text {Dist}}_{ij})$	-0.062
	(0.362)		(0.363)		(0.358)		(0.358)		(0.357)
N	54,250	N	54,250		59,950		59,950		59,950
Province-year effects	Yes	Province-time effects	Yes	Province-time effects	Yes	Province-time effects	Yes	Province-time effects	Yes
Country-year effects	Yes	Country-region effects	Yes	Country-region effects	Yes	Country-region effects	Yes	Country-region effects	Yes
Region-country effects	Yes	Country-time effects	Yes	Country-time effects	Yes	Country-time effects	Yes	Country-time effects	Yes

PPML estimates. Standard errors clustered at the province-country level in parentheses $$*$$$$p<0.1$$, $$**$$$$p<0.05$$, $$***$$$$p<0.01$$

### C.2 The geography of migration networks

As anticipated in Sect. 2, the networks of relevance for migrant effects could be localized or could extend beyond the province level (Herander and Saavedra 2005; Bratti et al. 2014). The localized nature of knowledge spillovers should make a localized effect more likely than a wider-ranging one. To investigate this argument, in column (1) of Table 13 we report the results of one specification of our gravity model that includes two additional variables: ${\text {In}}(ImmiOut_{ijt})$, measuring the total stock of immigrants from country *j* living in provinces other than *i* at time *t*, and ${\text {In}}(EmiOut_{ijt})$, i.e., the total stock of emigrants registered in provinces other than *i* who had migrated to country *j* at time *t*. These variables could account for extra-province networks of immigrants and emigrants, on which a focal province could draw to detect and exploit new export opportunities.

The results in column (1) show that geographical proximity actually matters in conveying the pro-export effects of emigration networks. Somewhat in line with the findings by Herander and Saavedra (2005) in the US, a weakly significant pro-export effect of immigrants does emerge for province-level networks. On the other hand, the emigrant effect seems to be driven by both a localized and a country-wide component. This is an interesting and important specification of our main result about the pro-export effect of emigrants. As argued by Rauch (2001), the exchange of trade-relevant information occurs mainly within networks of proximity. Indeed, this also allows the exchange of a tacit and embodied kind of trade-related information. On the other hand, a significantly positive and much larger effect emerges from ${\text {In}}(EmiOut_{ijt-1})$. This result suggests that the exports of a given province *i* to a country *j* rely not only on the pro-trade effects of emigrants from those provinces but also on emigrants from provinces other than *i* who live in *j*. To illustrate, exports from the province of Alicante to China increase not only with a larger network of Alicantinos moving to China but also with larger stocks of emigrants moving to China from Spanish provinces other than Alicante. This effect could be driven by a composite network effect, operating between migrants from different provinces meeting in the same foreign country, or by the preferences of expatriates from a given province for Spanish products as a whole, including from provinces other than that of their origin. Once again, both preference and information effects could be at play and a distinction between the two is, unfortunately, impossible with our available data. The different scales of the networks through which immigrants and emigrants exert their pro-export effect in the case of Spanish provinces—as previously mentioned, local and non-local, respectively—represents a novel result on which future research should focus.

### C.3 Concentration of immigration and emigration within provinces

Another implication of the full recognition of the role of geography in affecting migrant pro-trade effects is the need to allow their effects to be heterogeneous depending on their province-level concentration, as suggested in contributions by Herander and Saavedra (2005) and Peri and Requena-Silvente (2010).

Herander and Saavedra (2005) address the issue of the concentration of immigrants (not emigrants) within regions from the point of view of the spillovers that these could generate within co-ethnic networks. Specifically, they define as high-concentration regions those that hosted more than 10% of the foreign residents from a specific origin country. Their results indicate that information spillover effects occur from high-concentration regions into other regions.

Peri and Requena-Silvente (2010) study the heterogeneity of their results based on the overall immigration rates of each province, splitting their sample between provinces with immigration shares $< 4\%$, between 4 and 10%, and above 10% in 2007, which approximately corresponds to the tertiles of the distribution of immigration rates by province. Their results indicate that immigrants exert stronger pro-trade effects in provinces with higher shares of immigrant, consistent with the interpretation that immigration effects require some minimum share of the immigrant population to occur.

In our application, the presence of emigrants along with immigrants and of province-time fixed effects may make a difference with respect to the above findings. Moreover, strictly speaking, both studies apply a partial definition of concentration, considering that a standard concentration index of immigrants or emigrants would be obtained as a location quotient:

$$\begin{aligned} C_{ijt}^k=(\frac{n_{ijt}^k}{n_{jt}^k})/(\frac{n_{it}^k}{n_t^k}), \end{aligned}$$

where $k=\{m, e\}$, with *m* standing for immigrants and *e* for emigrants. When $k=m$, $n_{ijt}^m$ is the stock of immigrants from country *j* residing in province *i* in year *t*; $n_{jt}^m$ is the number of immigrants from country *j* residing nationwide in year *t*, $n_{it}^m$ is the number of immigrants from any country residing in province *i* in year *t*, and $n_{t}^m$ is the number of immigrants from any country residing nationwide in year *t*. When $k=e$, the index similarly refers to the concentration of emigrants from province *i* residing in country *j* at time *t*. Hence, a standard concentration index in a model that includes province-time fixed effects would be similar to a ratio of the indices used by Herander and Saavedra ($\frac{n_{ijt}^k}{n_{jt}^k}$) and by Peri and Requena-Silvente ($\frac{n_{it}^m}{{\text {population}}_{it}})$. Given that our model includes province-time fixed effects, however, the results should not be dramatically different whether we employ the definition by Herander and Saavedra or a location quotient.

The outcomes of the resulting analysis are reported in Table 13.

In columns (2)–(3), we analyze the presence of information spillover effects from high-concentration provinces. Following Herander and Saavedra (2005), in column (2), beyond immigration and emigration stocks, we include variables intended to capture the role of immigration and emigration in high- and low-concentration provinces. Specifically, ${\text {In}}(Immi_{ijt}^{HiConc}+1)$ is the log of the total stocks of immigrants from country *j* residing at time *t* in provinces where $\frac{n_{ijt}^m}{n_{jt}^m} > 10\%$ (to which we subtract bilateral stocks $Immi_{ijt}$ if *i* is a high-concentration province); ${\text {In}}(Immi_{ijt}^{LoConc}+1)$ represents the log of immigrants from country *j* residing in provinces where $\frac{n_{ijt}^m}{n_{jt}^m} \le 10\%$ (to which we subtract bilateral stocks $Immi_{ijt}$ if *i* is a low-concentration province); similarly, ${\text {In}}(Emi_{ijt}^{HiConc}+1)$ is the log of the total stocks of emigrants residing in country *j* that originate from provinces where $\frac{n_{ijt}^e}{n_{jt}^e} > 10\%$; and ${\text {In}}(Emi_{ijt}^{LoConc}+1)$ is the log of emigrants residing in country *j* who originate from provinces $\frac{n_{ijt}^e}{n_{jt}^e} \le 10\%$. Also in this case, the total stocks are net of bilateral stocks. According to Herander and Saavedra, the threshold of 10% allows for distinguishing the small set of provinces that concentrate the majority of residents from a specific country; in our data, this indeed identifies the upper 5% of the distribution of the ratio $\frac{n_{ijt}^m}{n_{jt}^m}$. Given that the distribution of $\frac{n_{ijt}^m}{n_{jt}^m}$ is similar to that of $\frac{n_{ijt}^e}{n_{jt}^e}$, we set the same threshold to identify provinces with a high concentration of emigrants.

In column (3), we perform the same exercise but change the classification of high- vs. low-concentration provinces. Here, high-concentration provinces are defined based on whether the location quotients $C_{ijt}^m$ and $C_{ijt}^e$ exceed their upper 5% value. In this way, we rescale our shares by the overall migrant stocks in the province relative to the whole country.

The two specifications yield very similar results. In both cases, immigrant and emigrant coefficients turn out to be positive and significant, with emigrant effects being larger in magnitude and more precisely estimated. In both cases, ${\text {In}}(Immi_{ijt-1}^{HiConc}+1)$ is positive and significant, while ${\text {In}}(Immi_{ijt-1}^{LoConc}+1)$ is insignificant. This indicates positive spillover effects from the immigrants residing in high-concentration provinces and no effects from those in low-concentration provinces. This is remarkably in line with the results by Herander and Saavedra. Instead, the coefficients of both ${\text {In}}(Emi_{ijt-1}^{HiConc}+1)$ and ${\text {In}}(Emi_{ijt-1}^{LoConc}+1)$ are insignificant.

Columns (4) and (5) are inspired by the specification by Peri and Requena-Silvente. To split the sample into tertiles of provinces with low, medium, and high immigration rates, we update the cutoffs to 5% and 11% so as to account for the growth in immigration rates compared with the earlier time period. As for emigration rates (columns 5–6), the tertiles are approximately identified by 1.5% and 3%. To study the moderating role of province- level immigration rates, we interact our log migration stocks with dummy variables indicating the tertile of the immigration rates that corresponds to each province. In so doing, we maintain the initial choice of including mutually exclusive interaction effects to facilitate the interpretation of the PPML estimates of the interaction effects.

The results confirm the overall findings in the paper and indicate a pattern that is compatible with the one identified in Peri and Requena-Silvente, yet less precise. Indeed, the effect of immigrants is insignificant in all provinces except in high-immigration ones. While the latter is only the case at the 10% significance level, it is compatible with the arguments about the need for a minimum level of immigration from any and all countries for the pro-trade effects to emerge. Larger networks of immigrants from any and all countries are likely to provide more services for immigrants (e.g., export intermediaries, call centers, cultural associations) that facilitate the emergence of pro-trade effects. Similar results, but more precise, are identified for emigration. Indeed, the effect of emigrants from low-emigration provinces is insignificant, while that of emigrants from medium- and high-emigration provinces is positive and highly significant, with a larger coefficient for high-emigration provinces. We could interpret this result similarly to that of immigration, arguing that larger emigration shares increase the services and knowledge available to expatriates that allow maintaining easier contact with the home provinces and facilitating trade. Given the asymmetry between immigrant and emigrant effects, however, the stronger effect of expatriates in provinces with high emigration rates may be driven by preference effects. In interpreting these results, we should remark that our empirical approach allows us to appreciate it net of the presumably lower overall export capacity of the provinces with very high emigration rates.

Overall, these results confirm that the concentration of immigrants and emigrants plays a role in facilitating the information flows that promote trade. We obtain similar but noisier results when studying the heterogeneity of the results regarding whether $C^k_{ijt}$ exceeds unity.

Interestingly, the immigrant effect that emerges can largely be ascribed to the few provinces that host the highest concentrations and shares of immigrants, which is found to trigger information spillover effects. As for emigrants, the effects turn out to be more spread across provinces.

**Table 13:** Geographic proximity and concentration of migrants within provinces

Geographic proximity		Spillover effects from high-concentration provinces			Heterogeneity by migration rates	
	(1)		(2)	(3)		(4)
$${\text {In}}({\text {Immi}}_{ijt-1}+1)$$	0.086$$*$$	$${\text {In}}(Immi_{ijt-1}+1)$$	0.062$$*$$	0.092$$**$$	$${\text {In}}(Immi_{ijt-1}^{<5\%}+1)$$	0.076
	(0.052)		(0.037)	(0.046)		(0.047)
$${\text {In}}({\text {ImmiOut}}_{ijt-1})$$	0.601	$${\text {In}}(Immi_{ijt-1}^{HiConc}+1)$$	0.055$$**$$	0.055$$**$$	$${\text {In}}(Immi_{ijt-1}^{5-11\%}+1)$$	0.044
	(0.492)		(0.023)	(0.027)		(0.041)
$${\text {In}}( {\text {Emi}}_{ijt-1}+1)$$	0.138$$***$$	$${\text {In}}(Immi_{ijt-1}^{LoConc}+1)$$	$$-$$0.418	0.491	$${\text {In}}(Immi_{ijt-1}^{>11\%}+1)$$	0.060$$*$$
	(0.042)		(0.451)	(0.401)		(0.035)
$${\text {In}}( {\text {EmiOut}}_{ijt-1})$$	1.221$$**$$	$${\text {In}}(Emi_{ijt-1}+1)$$	0.087$$**$$	0.115$$***$$	$${\text {In}}(Emi_{ijt-1}^{<1.5\%}+1)$$	0.048
	(0.618)		(0.037)	(0.039)		(0.043)
$${\text {NI}}_{ijt-1}$$	-0.110	$${\text {In}}(Emi_{ijt-1}^{HiConc}+1)$$	$$-$$0.005	$$-$$0.023	$${\text {In}}(Emi_{ijt-1}^{1.5-3\%}+1)$$	0.093$$**$$
	(0.099)		(0.009)	(0.068)		(0.038)
$${\text {NE}}_{ijt-1}$$	0.003	$${\text {In}}(Emi_{ijt-1}^{LoConc}+1)$$	$$-$$0.391	0.213	$${\text {In}}(Emi_{ijt-1}^{>3\%}+1)$$	0.119$$***$$
	(0.066)		(0.485)	(0.135)		(0.039)
${\text {In}}( {\text {Dist}}_{ij})$	$-$0.018	${\text {In}}( {\text {Dist}}_{ij})$	0.018	$-$0.029	${\text {In}}( {\text {Dist}}_{ij})$	0.053
	(0.356)		(0.360)	(0.349)		(0.362)
N	54,250	N	54,250	53,811		54,250
Province-year effects	Yes	Province–time effects	Yes	Yes	Province-time effects	Yes
Country-year effects	Yes	Country-region effects	Yes	Yes	Country-region effects	Yes
Region-country effects	Yes	Country-time effects	Yes	Yes	Country-time effects	Yes

PPML estimates. Standard errors clustered at the province-country level in parentheses $$*$$$$p<0.1$$, $$**$$$$p<0.05$$, $$***$$$$p<0.01$$.

Column (2): HiConc $=\frac{n_{ijt}^m}{n_{jt}^m}>10\%$; Column (3): HiConc $=C^m_{ijt}>95{\mathrm{th}}$ percentile

### C.4 Migration–trade links and province-level production of intermediate goods

According to the arguments by Rauch (1999) and Rauch and Trinidade (2002), we expect the information effects of immigrants to be stronger for the trade of goods with stronger informational content, i.e., differentiated goods. The export of differentiated goods may also be more sensitive to emigrant preference effects: among the available varieties, the presence of origin-culture-specific features may increase the probability that expats select a particular variety over another, even when purchasing intermediate goods (Nefussi and Schwellnus 2010). In contrast, according to Rauch and Trinidade, the trade in homogeneous and reference-priced goods is less sensitive to immigrant information effects and requires less intermediation. This is so given that prices convey most of the relevant information for the trade of these goods. Hence, overall, we expect stronger immigrant and emigrant pro-trade effects in the trade of differentiated rather than homogeneous goods.

Rauch’s original classification is based on the 4-digit SITC classification of goods. Lacking complete data on bilateral Spanish exports by type of good, we can only address the issue with some degree of approximation. We draw on publicly available data on the exports of Spanish provinces by sector from DataComex.^34^ Exports in intermediate manufactures are more likely to fall into the homogeneous and reference-priced categories (Burchardi et al. 2018). Among these, we select the exports in the energy sector, raw materials, and semi-finished products and exclude capital goods and machineries that are, on the contrary, highly customized and differentiated. We compute a location quotient for the production of these goods by the provinces in our sample. The underlying idea is that immigrant and emigrant effects in provinces that are specialized in the export of these products are more likely to concern the mediation of trade transactions in these sectors.

Specifically, we compute the location quotient for the export of intermediate goods in the energy sector, raw materials, and semi-finished products for each province and year between 2007 and 2016; we average them over the entire period and select the 10 provinces with the highest average location quotient: Asturias, Bizkaia, Burgos, Castellon, Cadiz, Huelva, Lugo, Las Palmas, Santa Cruz de Tenerife, and Tarragona. We label them “intermediate goods exporting” (IGE) for simplicity. We then study the heterogeneity of our results along this margin and the robustness of our results to the exclusion of the IGE provinces.

The results are reported in Table 14. In general, they support our main results in that the main pro-export effects are observed for emigrants. Moreover, we find that the effect is entirely driven by emigrants coming from provinces that are not specialized in intermediate sectors. Immigrants from non-IGE provinces turn out to be positive and significant, although only at the 10% level. When excluding the top 10 IGE provinces, the results for emigrants become more noisy but are still significant at the 6% level, and the results for immigrants increase in magnitude and significance. In conclusion, our results are in line with the arguments by Rauch about a stronger effect of migration for more differentiated goods and with the hypothesis that preference effects may also be stronger in this regard. While the main results of our analysis are supported, the emerging effect of immigration for the subsample of non-IGE provinces suggests that pooling different sectors may have led to some loss of information and that the heterogeneity along this line should be addressed in further research with more detailed data.

**Table 14:** Migration–trade links and province-level production of intermediate goods

Sample excluding IGE provinces		Heterogeneity by IGE	
	(1)		(2)
$${\text {In}}({\text {Immi}}_{jit-1}+1)$$	0.082$$**$$	$${\text {In}}({\text {Immi}}^{\mathrm{IGE}}_{jit-1}+1)$$	0.047
	(0.041)		(0.038)
		${\text {In}}({\text {Immi}}^{\mathrm{not IGE}}_{jit-1}+1)$	0.063$*$
			(0.037)
${\text {In}}({\text {Emi}}_{jit-1}+1)$	0.092$*$	${\text {In}}({\text {Emi}}^{\mathrm{IGE}}_{jit-1}+1)$	0.059
	(0.047)		(0.040)
		$${\text {In}}({\text {Emi}}^{\mathrm{not IGE}}_{jit-1}+1)$$	0.112$$***$$
			(0.037)
N	43,400	N	54,250

All specifications include province-time, country-time, and region-country

fixed effects, as well as log distance. IGE=intermediate good exporting,

as explained in the text. Standard errors clustered at the pair level in parentheses.

$$*$$$$p<0.1$$, $$**$$$$p<0.05$$, $$***$$$$p<0.01$$

Overall, while some heterogeneity in the effects emerges, the results of our estimators are similar to each other and do not raise substantial concerns that the larger weight given to larger observations by the PPML is biasing the results.

### C.5 Additional results tables

See Tables 15, 16 here.

**Table 15:** Park test

	(1)
$${\text {In}}({\text {Immi}}_{ijt-1}+1)$$	0.291$$***$$
	(0.038)
$${\text {In}}({\text {Emi}}_{ijt-1}+1)$$	0.540$$***$$
	(0.051)
$${\text {NI}}_{ijt-1}$$	$$-$$0.348$$***$$
	(0.112)
${\text {NE}}_{ijt-1}$	$-$0.062
	(0.078)
$${\text {In}}({\text {Dist}}_{ij})$$	$$-$$3.311$$***$$
	(0.795)
N	50,210
Province-year effects	Yes
Region-country effects	Yes
Country-year effects	Yes

Standard errors clustered at the province-country level in parentheses

$$*$$$$p<0.1$$, $$**$$$$p<0.05$$, $$***$$$$p<0.01$$

**Table 16:** Immigrant effects over time

	Coef. (SE)		Coef. (SE)
$${\text {In}}({\text {Immi}}_{ijt-1}+1) \times ({\text {year}}=2007)$$	0.076$$**$$	$${\text {In}}({\text {Emi}}_{ijt-1}+1) \times ({\text {year}}=2007)$$	0.053
	(0.036)		(0.039)
$${\text {In}}({\text {Immi}}_{ijt-1}+1) \times ({\text {year}}=2008)$$	0.078$$**$$	$${\text {In}}({\text {Emi}}_{ijt-1}+1) \times ({\text {year}}=2008)$$	0.080$$**$$
	(0.037)		(0.041)
$${\text {In}}({\text {Immi}}_{ijt-1}+1) \times ({\text {year}}=2009)$$	0.086$$**$$	$${\text {In}}({\text {Emi}}_{ijt-1}+1) \times ({\text {year}}=2009)$$	0.078$$*$$
	(0.039)		(0.042)
${\text {In}}({\text {Immi}}_{ijt-1}+1) \times ({\text {year}}=2010)$	0.062	${\text {In}}({\text {Emi}}_{ijt-1}+1) \times ({\text {year}}=2010)$	0.070$*$
	(0.038)		(0.041)
${\text {In}}({\text {Immi}}_{ijt-1}+1) \times ({\text {year}}=2011)$	0.053	${\text {In}}({\text {Emi}}_{ijt-1}+1) \times ({\text {year}}=2011)$	0.057
	(0.042)		(0.041)
$${\text {In}}({\text {Immi}}_{ijt-1}+1) \times ({\text {year}}=2012)$$	0.049	$${\text {In}}({\text {Emi}}_{ijt-1}+1) \times ({\text {year}}=2012)$$	0.101$$**$$
	(0.042)		(0.041)
$${\text {In}}({\text {Immi}}_{ijt-1}+1) \times ({\text {year}}=2013)$$	0.035	$${\text {In}}({\text {Emi}}_{ijt-1}+1) \times ({\text {year}}=2013)$$	0.149$$***$$
	(0.043)		(0.044)
$${\text {In}}({\text {Immi}}_{ijt-1}+1) \times ({\text {year}}=2014)$$	0.025	$${\text {In}}({\text {Emi}}_{ijt-1}+1) \times ({\text {year}}=2014)$$	0.135$$***$$
	(0.040)		(0.043)
$${\text {In}}({\text {Immi}}_{ijt-1}+1) \times ({\text {year}}=2015)$$	0.050	$${\text {In}}({\text {Emi}}_{ijt-1}+1) \times ({\text {year}}=2015)$$	0.128$$***$$
	(0.041)		(0.043)
$${\text {In}}({\text {Immi}}_{ijt-1}+1) \times ({\text {year}}=2016)$$	0.033	$${\text {In}}({\text {Emi}}_{ijt-1}+1) \times ({\text {year}}=2016)$$	0.119$$***$$
	(0.040)		(0.044)

$N=54,250$. Included but unshown covariates are: ${\text {NI}}_{ijt-1}$, ${\text {NE}}_{ijt-1}$, ${\text {In}}({\text {Dist}}_{ij})$,

as well as province-year, country-year, and region-country effects. PPML estimates.

Standard errors clustered at the province-country level in parentheses.

$$*$$$$p<0.1$$, $$**$$$$p<0.05$$, $$***$$$$p<0.01$$

## Footnotes

1 See, for instance, the “Migration & Economics” Virtual Issue by Oxford University Press, available at https://www.global.oup.com/academic/category/social-sciences/economics/economics_of_migration.

2 For a review of this literature, see Felbermayr et al. (2015). Recent advancements include Burchardi et al. (2018), Parsons and Vézina (2018), and Bratti et al. (2020).

3 The list of subnational units adopted for the analysis of the migration–trade link includes: US states (Herander and Saavedra 2005; Dunlevy 2006; Bandyopadhyay et al. 2008; Burchardi et al. 2018), Canadian provinces (Wagner et al. 2002), Italian provinces (Bratti et al. 2014, 2020), French departments (Briant et al. 2014), and Spanish provinces (Peri and Requena-Silvente 2010).

4 Ceuta and Melilla are excluded due to reasons of data availability.

5 The first contributions date back to Gould (1994); Head and Ries (1998); Rauch and Trinidade (2002).

6 Notable exceptions are the country-level studies by Murat and Pistoresi (2009); Parsons (2012); Hiller (2014); Felbermayr et al. (2015).

7 In general, $Y_{i}$ and $Y_{j}$ represent the “mass” of production and the “mass” of expenditures of the exporting and the importing partner, respectively, and are proxied by their GDP. $\phi _{ij}$ instead refers to natural trade barriers, such as the geographical distance between countries, but also to other economic barriers, such as tariffs, as well as to their respective elasticities.

8 More precisely, $\Omega _i$ represents the “expenditure-weighted average of relative access” and $\Phi _j$ the “accessibility-weighted sum of exporters’ capabilities” (Head and Mayer 2014, : 140–141).

9 As Head and Mayer (2014) have shown in their review, “structural” gravity equations (and their “general” form) are compatible with the wide majority of trade models used in the literature.

10 In their framework, based on Arkolakis et al. (2012), the subnational exporting capacity is modeled as a function of the number of exporters in province *i* and on the price charged by the exporters for their varieties. In turn, the price charged by province *i* exporters for products exported to *j* depends on production costs (i.e., wages), transportation costs, and the productivity of *i* firms exporting to *j* relative to the average productivity of firms in *i*.

11 Similarly, the importer-side MRT should be seen as the average (weighted) market access of a given country to any province in Spain, as well as to any other exporter worldwide.

12 It should be noted that we include distance as a regressor mainly as a control variable to mitigate the effects of residual dyadic heterogeneity, in the spirit of Baier and Bergstrand (2007), and not to study the effects of distance *per se*. Of course, the effects of distance will be largely absorbed by the region-country fixed effects. The estimated coefficient of distance will only capture the extent to which a given province is more or less distant from the partner country than the average of the region. Hence, it will not be particularly insightful. Reassuringly, our results are fully robust to excluding distance, which we interpret as an indication that residual pair heterogeneity does not affect our estimates (see Table 9 in the Appendix).

13 In the case of OLS, the test reduces to estimating ${\text {ln}}\hat{\epsilon }^{2}_{ij}={\text {constant}}+\lambda \widehat{{\text {ln}}X}_{ij}$.

14 This, and their similar first-order conditions, explains why the Gamma and OLS estimates are often quite similar (Head and Mayer 2014).

15 https://www.ine.es/dyngs/INEbase/es/operacion.htm?c=Estadistica_C&cid=1254736177013&menu=resultados&idp=1254734710990#!tabs-1254736195469.

16 They instrument the total inflows of immigrants to a given province by aggregating bilateral flows estimated through a gravity model of international migration.

17 In this application, we will not implement the standard error correction for shift-share instruments proposed by Adao et al. (2019) or the related approach by Borusyak et al. (2018). These techniques do not allow us to include two instrumental variables based on different shares and would thus not allow us to compare the effects of immigration with those of emigration. Furthermore, these approaches require an assumption of many independent shocks affecting each pair, which does not seem appropriate with our modified shift-share instruments. As previously mentioned, in our case the imputed values of immigration and emigration for each pair rely on a single share and a single shifter each.

18 All inference reported in this section is based on standard errors that are clustered at the pair level. The results are robust to multi-way clustering of the errors at the province-year, country-year, and region-country levels as well as at the province and country levels.

19 In practice, the regression output of some applications—but not in our case—may still yield a coefficient for this variable, but this will refer to the coefficient for the reference category of the regression, which will typically not be under the analyst’s control when high-dimensional fixed effects are included. Hence, Head and Mayer (2014) recommend omitting these terms.

20 We leave to future research the further exploration of the sources of misspecification in the GPML model, which do not seem to be driven by the incidental parameters problem (Greene 2004; Weidner and Zylkin 2020) or by functional form misspecification. According to Santos-Silva and Tenreyro (2006), the reason may be found in the larger weight that the GPML gives to smaller observations.

21 We exclude the log of distance from these estimates as it leads to rank deficiency.

22 Data on pre-determined stocks and flows are available for a slightly smaller subset of trade pairs than the official trade data, and this leads to a small reduction in the number of observations. Results for this smaller set of dyads are fully robust and are thus unreported.

23 Interestingly, the 2SLS estimate of our emigrant effect is closer than our baseline estimate to the immigration elasticities of imports detected by Bratti et al. (2014).

24 The Hansen J test similarly does not reject the null when run on a specification that only includes immigrants as an endogenous variable ($\chi ^2(1)$= 0.000,* p*-value = 0.9924).

25 It is well known that the magnitude of the standard interaction effects in non-linear models does not equal the marginal effects of the interaction between the two (Ai and Norton 2003). Therefore, throughout this paper and for ease of interpretation, we do not report the standard main effect along with the interaction effects, but rather a set of mutually exclusive interaction terms for the variables under scrutiny. The identified patterns correspond to that of the migration elasticities calculated algebraically on the basis of the estimated marginal effects of the interaction terms.

26 In brief, this model investigates the probability that given a set of realizable trade opportunities, the migrant actually realizes them.

27 We thank an anonymous reviewer for suggesting this analysis.
